# Supplementary material for: Staff experiences of enhanced recovery after surgery: systematic review of qualitative studies
Source: BMJ Open. 2019 Feb 12;9(2):e022259. doi: 10.1136/bmjopen-2018-022259 (PMC6377558; doi:10.1136/bmjopen-2018-022259)
Supplement: Supplementary data [file bmjopen-2018-022259supp001.pdf]

PRISMA 2009 Flow Diagram
Staff experiences of Enhanced Recovery after Surgery – Systematic Review of Qualitative Studies

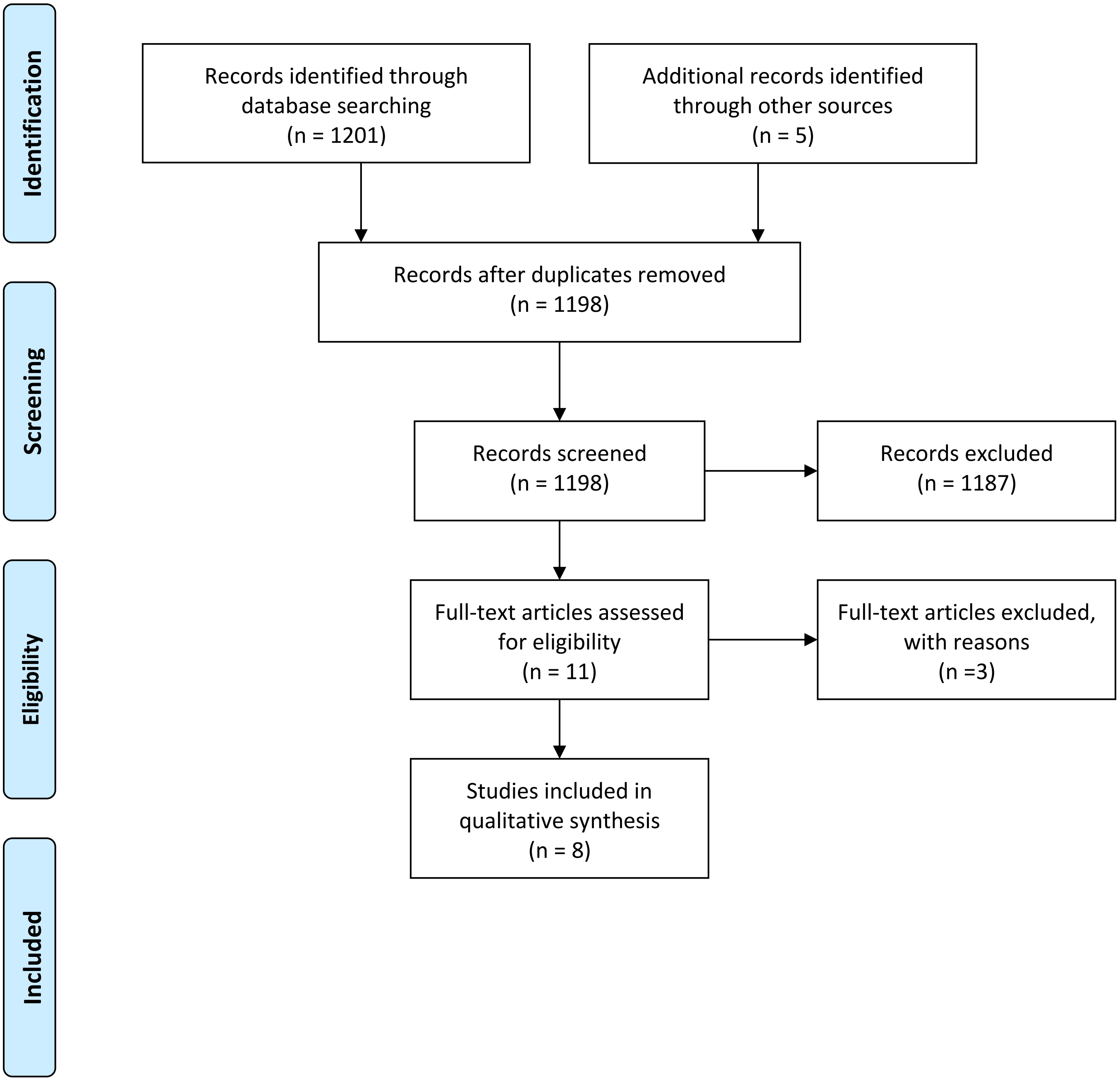

From: Moher D, Liberati A, Tetzlaff J, Altman DG, The PRISMA Group (2009). *P*referred *R*eporting *I*tems for *S*ystematic Reviews and *M*eta-*A*nalyses: The PRISMA Statement. PLoS Med 6(7): e1000097. doi:10.1371/journal.pmed1000097

For more information, visit www.prisma-statement.org.
