# Supplementary material for: Staff experiences of enhanced recovery after surgery: systematic review of qualitative studies
Source: BMJ Open. 2019 Feb 12;9(2):e022259. doi: 10.1136/bmjopen-2018-022259 (PMC6377558; doi:10.1136/bmjopen-2018-022259)
Supplement: Author's manuscript [file bmjopen-2018-022259.draft_revisions.pdf]

BMJ Open

BMJ Open is committed to open peer review. As part of this commitment we make the peer review history of every article we publish publicly available.

When an article is published we post the peer reviewers' comments and the authors' responses online. We also post the versions of the paper that were used during peer review. These are the versions that the peer review comments apply to.

The versions of the paper that follow are the versions that were submitted during the peer review process. They are not the versions of record or the final published versions. They should not be cited or distributed as the published version of this manuscript.

BMJ Open is an open access journal and the full, final, typeset and author-corrected version of record of the manuscript is available on our site with no access controls, subscription charges or pay-per-view fees (http://bmjopen.bmj.com).

If you have any questions on BMJ Open's open peer review process please email info.bmjopen@bmj.com

BMJ Open

Staff Experiences of Enhanced Recovery after Surgery – Systematic Review of Qualitative Studies

Journal:	*BMJ Open*
Manuscript ID	bmjopen-2018-022259
Article Type:	Research
Date Submitted by the Author:	23-Feb-2018
Complete List of Authors:	Cohen, Rachel; University of Bristol, School of Population Health Sciences<br>Gooberman-Hill, Rachael; University of Bristol, School of Clinical Sciences
Keywords:	Enhanced recovery, Qualitative, Joint replacement

SCHOLARONE™
Manuscripts

For peer review only

Article Title: STAFF EXPERIENCES OF ENHANCED RECOVERY AFTER SURGERY – SYSTEMATIC REVIEW OF QUALITATIVE STUDIES

Authors: [#]Dr Rachel Cohen and Professor Rachael Gooberman-Hill*

***Corresponding author:** Dr Rachel Cohen, Centre for Academic Mental Health, Department of Population Health Sciences, Bristol Medical School, University of Bristol, Bristol, BS8 2BN, UK

Corresponding author's Email address: rachel.cohen@bristol.ac.uk Tel: +44(0)117 331 4007

[#]Co-author: Professor Rachael Gooberman-Hill, Professor of Health and Anthropology, Translational Health Sciences, Bristol Medical School, University of Bristol, Learning and Research Building, Level 1, Southmead Hospital, Bristol BS10 5NB, UK

Word count: 4194

Keywords: Enhanced Recovery, qualitative, joint replacement

For peer review only

For peer review only - http://bmjopen.bmj.com/site/about/guidelines.xhtml

ABSTRACT

Objective: To conduct a systematic review of qualitative studies which explore health professionals' experiences of and perspectives on the enhanced recovery after surgery (ERAS) pathway.

Design: Systematic review of qualitative literature using a qualitative content analysis.

Setting: Secondary care, including a wide range of hospital settings.

Participants: Health professionals including a wide range of Multidisciplinary Team and Allied Health Professional staff to incorporate a diverse range of clinical and professional perspectives.

Results: Eight studies were included in the review. These studies focus on the implementation and delivery of ERAS across a range of clinical contexts, including colorectal surgery, gastrointestinal surgery, abdominal hysterectomy, and orthopaedics. Health professionals participating in the studies include registrars, consultants, surgeons, anaesthetists, doctors, nurses and physiotherapists, nursing managers, ERAS coordinators and champions, care coordinators and service improvement coordinators. Five main themes emerged in the analysis: communication and collaboration, resistance to change, role and significance of protocol-based care, knowledge and expectations, and temporality. Professionals described the importance of effective multidisciplinary team collaboration and communication, providing thorough education to staff and patients, and appointing a dedicated champion as means to implement and integrate ERAS pathways successfully. Evidence based protocol based guidelines were thought to be useful for improvements to patient care by standardising practices and reducing treatment variations, but were thought to be too open to interpretation at local levels. Setting and managing 'realistic' expectations of staff was seen as a priority. Staff attitudes towards ERAS tend to become more favourable over time, as practices become successfully 'normalised'.

Conclusions: Staff feel positive about the implementation of ERAS, but acknowledge that the process is complex and challenging. Many of the challenges identified, such as resistance to change and lack of confidence can however be mitigated by ensuring that multidisciplinary teams understand ERAS principles and guidelines, and that they communicate well with one another and

For peer review only - http://bmjopen.bmj.com/site/about/guidelines.xhtml

with patients. We suggest that the provision of comprehensive, coherent and locally relevant information to health professionals. Identifying and recruiting local ERAS champions would help to improve the implementation and delivery of ERAS pathways.

ARTICLE SUMMARY

Strengths and limitations of this study

- There are few existing systematic reviews of qualitative studies on staff experiences of ERAS
- The review includes studies with a diverse range of populations, contexts and methodological approaches
- The review includes a small number of studies, reducing its transferability

For peer review only

INTRODUCTION

Enhanced recovery after surgery (ERAS) programmes were introduced and began to be implemented in the late 1990s[1], as part of an initiative towards reducing variations in patient care and improving quality standards[2]. Building upon their Danish origins, ERAS programmes have been internationally adopted, and widely implemented for major elective surgical pathways in colorectal surgery, orthopaedics, gynaecology, cardiology and urology. Depending upon the kind of diagnostic and surgical care in question, ERAS programmes are sometimes referred to using different names, including 'fast-track surgery', 'rapid recovery', 'accelerated discharge' or 'early discharge'.

The aim of ERAS pathways is to reduce length of hospital stay and lessen readmissions, minimise surgical complications, decrease morbidity, and improve cost effectiveness. Best described as a complex intervention[3,4], ERAS seeks to improve patient experiences and outcomes by focusing on key aspects of the care pathway, pre-, peri- and post operatively, as a means of reducing physiological and psychological stress. This involves the provision of better education and information for patients prior to their operations, the use of minimally invasive surgical techniques and anaesthesia, optimal pain management and early post-operative mobilisation, as well as the preparation of a discharge plan[5].

Despite their protocol-based foundations, evidence from recent studies indicates that ERAS pathways are implemented variably across different hospital settings. More information is needed about what the core active ingredients of ERAS are. We also need to know more about how these ingredients exert their effect according to local circumstances, and about how they shape (and are shaped by) the context of their implementation[4,6]. Existing literature has drawn particular attention to the factors which help, and those which hinder, the successful implementation of ERAS, identifying important barriers and facilitators to the process Barriers include resistance to change, inadequate funding, lack of support from management, high staff turnover, poor documentation,

For peer review only - http://bmjopen.bmj.com/site/about/guidelines.xhtml

and shortness of time, whilst facilitators included a dedicated enhanced recovery lead, effective multidisciplinary team working and ongoing education for staff and patients[7].

Patient experiences of and satisfaction with ERAS pathways have been studied using both quantitative and qualitative approaches: the latter have been especially useful in improving understandings of patient experiences and perspectives e.g.[8-13]. Sibbern et al's[14] systematic review of such studies provides a comprehensive discussion of existing qualitative research on this specific topic. Health professionals' satisfaction with and perspectives on ERAS, meanwhile, have typically been explored using quantitative approaches. Information regarding the experiences of health professionals in this context is needed to inform ongoing healthcare policy and practice.

This article describes a systematic review of qualitative studies of health professionals' experiences of ERAS pathways. By synthesising existing research the review aims to identify overarching themes that provide opportunities for improving implementation and practice.

METHODS

Patient and Public Involvement

This paper is a systematic review of qualitative studies. No patients were involved in the review.

Our systematic review was registered on PROSPERO in 2017: the registration number is CRD42017059952. The review sought to describe the experiences and perspectives of healthcare professionals involved in delivering enhanced recovery pathways.

We used methods of systematic search and review and conducted a search of PsychINFO, Medline, Cinahl, and PubMed to identify relevant qualitative studies across a range of health care contexts. For all of the databases, the search terms used were:

For peer review only - http://bmjopen.bmj.com/site/about/guidelines.xhtml

ERAS OR enhanced recovery OR fast-track OR accelerated recovery OR rapid recovery OR early discharge OR patients discharge OR enhance* recov* after surg*

Staff perspective OR staff experience* OR staff perception* OR ward staff OR nurs* OR professional*

Qualitative OR interview* OR ethnograph* OR observation

The reference lists of articles identified from the database search were also scrutinised for possible additional studies.

As shown in the PRISMA flowchart (supplementary file), the database searches yielded 1201 articles in total. In addition, through searching the reference lists of the included studies, we identified five further records. Eleven studies met the inclusion criteria, and were assessed for eligibility using the Critical Appraisal Skills Programme (CASP) guidance (Critical Skills Appraisal Programme 2017)[15]. The CASP tool for systematic reviews provides a means of identifying the strengths and weaknesses of research articles, assessing their usefulness and validity, and their relevance for inclusion in the review. After considering the ten CASP domains – aim, methodology, design, recruitment strategy, data collection, relationship between researcher and participants, ethical issues, data analysis, findings and research value - three studies were excluded, and the remaining eight were included. Two of the three that were excluded at this stage were quantitative rather than qualitative, and one focused on rehabilitation following hip and knee arthroplasty, but not specifically on ERAS. The two authors independently conducted quality assessment and agreed that all eight articles addressed all ten CASP criteria and were of sufficient rigour and relevance for inclusion in the review.

After completion of quality assessment we conducted a qualitative meta-synthesis of the eight eligible articles. This comprised close reading and extraction of key findings using descriptive

For peer review only - http://bmjopen.bmj.com/site/about/guidelines.xhtml

For peer review only

qualitative design[16], and a qualitative content analysis[17,18]. For the analysis, we focused on the manifest content of the articles, i.e. what the texts say[17]. This involved searching for the common concepts and themes[18] addressed in the articles regarding health professionals' experiences of and perspectives on ERAS. Supporting quotes were also gathered. This enabled us to develop meaning units, extracted from the findings of the studies. These were then condensed into content related categories, which the authors discussed and agreed upon. We then synthesised the chosen categories into themes as shown in Table 1.

TABLE 1

Theme 1: Collaboration and communication

MEANING UNITS	CONTENT RELATED CATEGORIES
Staff find the information-rich nature of ERAS confusing. Many staff feel that they do not understand it well enough and/or that they have not received sufficiently clear or consistent information or training.	Providing staff and their patients with a comprehensive education about and introduction to ERAS improves understanding and helps to mitigate confusion.
Information about ERAS is not always disseminated between staff – and between staff and patients – in a coherent and consistent way.	Strong team communications help to ensure the effective dissemination of information
Collaborative MDT work is hindered by high staff turnover and a lack of coordination across different departments.	Building good relationships within the MDT helps to encourage dialogue between staff, and to improve their willingness and ability to collaborate. The appointment of a dedicated ERAS 'champion' improves staff engagement and collaborative working,

Theme 2: Resistance to change

For peer review only - http://bmjopen.bmj.com/site/about/guidelines.xhtml

MEANING UNITS	CONTENT RELATED CATEGORIES
Staff are reluctant to implement or engage with new and unfamiliar working practices. Some staff – especially those who are older, or more well established in their role – tend to dislike change more generally and are disinclined to engage with ERAS.	Appointing and ERAS champion helps to encourage more positive attitudes amongst staff.

THEME 3: Role and significance of protocol-based care

MEANING UNITS	CONTENT RELATED CATEGORIES
Staff recognise the usefulness of evidence-based protocol guidelines as a means of reducing variations and standardising practice, but have mixed feelings about whether ERAS facilitates this well.	The incorporation of standardised order sets and basing ERAS practices on best evidence increases staff willingness to implement it as a complex intervention.
ERAS is not definitively prescriptive, and therefore allows for too much variability in local implementation.	Having a local ERAS champion helps to improve consistency in implementing and operationalising the pathway into existing systems at local sites.
Some staff feel conflicted about having to compromise their capacity for and confidence in providing individualised care for patients.	Clearer guidance about when it is acceptable to deviate from ERAS protocols would improve staff confidence.

THEME 4: Knowledge and Expectations

MEANING UNIT	CONTENT RELATED CATEGORY
Staff feel that they need a broader knowledge and understanding of ERAS, i.e. beyond protocol guidelines.	Belief in the value and potential positive impact of ERAS improves the willingness of staff to engage with the pathway and its guidelines.
Staff are sceptical about the usefulness and value	Staff feel more positive about and favourable

For peer review only - http://bmjopen.bmj.com/site/about/guidelines.xhtml

of ERAS prior to its implementation.	towards ERAS when they have seen it work successfully in practice.
Managing the expectations of staff and patients is recognised as being crucial to the successful implementation of ERAS. Differing professional perspectives, which are sometimes based on incorrect assumptions, can create ambivalence and uncertainty amongst staff. Staff use tacit knowledge and a "common sense" approach to overcome this.	Setting clear and realistic expectations about ERAS helps to improve staff and patient experiences of the pathway.

THEME 5: Temporality

MEANING UNIT	CONTENT RELATED CATEGORY
The successful implementation and embedding of ERAS is a gradual process.	ERAS becomes "normalised" over time, and staff attitudes towards the pathway tend to become more positive. There are ongoing challenges with a lack of available resources (financial and administrative) that are necessary to successfully maintain ERAS over time.

For peer review only - http://bmjopen.bmj.com/site/about/guidelines.xhtml

FINDINGS

The eight studies included were conducted in the UK (n = 1), US (n = 1), Canada (n = 2), Denmark (n = 2), and Australia (n = 1) (Table 2).

TABLE 2

**CITATION**	**Study design and key findings**
Alawadi, Z.M., Leal, I., Phatak, U.R., Flores-Gonzalez, J.R., Holihan, J.L., Karanjawala, B.E., Millas, S.G. and Kao, L.S. (2016) Facilitators and barriers of implementing enhanced recovery in colorectal surgery at a safety net hospital: A provider and patient perspective. *Surgery* 159(3), 700-712	• Qualitative interviews with MDT hospital staff and patients: 'key stakeholders involved in receiving and implementing an ERAS pathway' (2016: 702)<br>• ERAS for colorectal surgery: study conducted in US<br>• Staff identified 5 facilitators and 5 barriers; patients identified 4 facilitators and 3 barriers<br>• Conclusion: 'Although limited hospital resources are perceived as a barrier to ERAS implementation… there is strong support for such pathways and multiple factors were identified that may facilitate change' (2016: 700)
Sjetne, I.S., Krogstad, U., Odegard, S. and Engh, M.E. (2009) Improving quality by introducing enhanced recovery after surgery in a gynaecological department: consequences for ward nursing practice. *Quality and Safety in Healthcare*, 18: 236-240	• Qualitative interviews with patients and nurses – four nurses were informants in a total of nine interviews<br>• ERAS for gynaecological surgery – study conducted in Norway<br>• Conclusion: 'expected clinical gains achieved by introducing ERAS are achieved without compromising the work environment of ward nurses' (2009: 239)
Pearsall, E.A., Meghiji, A., Pitzul, K.B., Aarts, M., McKenzie, M., McLeod, R.S. and Okrainec, A. (2015) A Qualitative Study to Understand the Barriers and Enablers in Implementing an Enhanced Recovery After Surgery Program. *Annals of Surgery* 261(1): 92-96	• Qualitative interviews with general surgeons, anaesthesiologists and ward nurses<br>• ERAS for colorectal surgery – study conducted in Canada<br>• Focus on perioperative care<br>• Useful paper because it breaks results down into overall, intervention-specific and discipline-specific barriers and enablers, giving a rich multidisciplinary perspective<br>• Conclusion: 'participants supported the need for implementation of an ERAS program… [but] felt there remained major barriers to [its] successful implementation' (2015: 96)

For peer review only - http://bmjopen.bmj.com/site/about/guidelines.xhtml

Wagner, L., Carlslund, A.M., Moller, C. and Ottesen, B. (2004) Patient and staff (doctors and nurses) experiences of abdominal hysterectomy in accelerated recovery programme: A qualitative study. *Danish Medical Bulletin* 51(4): 418-421	• Qualitative individual interviews and focus groups with staff, observation of and interviews with patients<br>• Accelerated recovery for abdominal hysterectomy – study conducted in Denmark<br>• Useful study because it explores staff experiences with (i) changes in care, (ii) staff perspectives on implementation and (iii) comparisons/contrasts between staff and patient expectations<br>• Conclusion: patients underwent ARP without significant problems, but identified a need for greater psychological support. Staff data showed a positive change in opinion and an understanding of ARP. Recommendations made for better information to be provided to staff and patients, in consultation rooms and outpatient clinics
Jeff, A. and Taylor, C. (2014) Ward nurses' experience of enhanced recovery after surgery: a grounded theory approach. *Gastrointestinal Nursing* 12(4): 23-31	• Qualitative interviews with 8 ward nurses (4 'experienced' and 4 'newer' to ERAS), as well as documentary evidence using memos and a reflective journal<br>• ERAS for gastrointestinal surgery – study conducted in the UK<br>• Focus on postoperative care<br>• Conclusion: 'the central difficulty experienced by nurses was trying to adapt the protocol to the demands of patient care delivery within the constraints of their role and organisational culture' (2014: 31)
Gotlib Conn, L., McKenzie, M., Pearsall, E.A. and McLeod, R.S. (2015) Successful implementation of an enhanced recovery after surgery programme for elective colorectal surgery: a process evaluation of champions' experiences. *Implementation Science* 10(1): 1-11	• Process evaluation: qualitative interviews with implementation champions, including surgeons, anaesthesiologists, nurses and project coordinators. 15 participating hospitals<br>• ERAS for elective colorectal surgery – study conducted in Ontario<br>• Paper is useful because it is a qualitative process evaluation, and uses Normalisation Process Theory for data analysis<br>• Conclusion: successful implementation of ERAS is achieved by a 'complex series of cognitive and social processes… [the study demonstrates the importance of] champion coherence, external and internal relationship building, and the strategic management of a project's organisation-level visibility' (2015: 1)
Lyon, A., Solomon, M.J., and Harrison, J.D. (2014) A Qualitative Study Assessing the Barriers to Implementation of Enhanced Recovery After Surgery. *World Journal of Surgery* 38: 1374-1380	• Includes in-depth, semi-structured interviews with staff including consultants, surgeons, nurses, physiotherapists, care coordinators and medical administrators<br>• ERAS for colorectal surgery – study conducted in Sydney<br>• Paper is useful because it focuses on the reasons behind compliance issues in ERAS, and assesses these qualitatively. It also recognises that understanding the views of a range of MDT staff is important<br>• Study shows that there are four key areas that present barriers to successful ERAS implementation: (i) patient-

For peer review only - http://bmjopen.bmj.com/site/about/guidelines.xhtml

	related factors, (ii) staff-related factors, (iii) practice-related issues and (iv) resources. Paper provides a very comprehensive discussion of these themes<br>• Conclusion: for ERAS to be implemented successfully and function efficiently with high levels of compliance, these key areas need to be addressed (ideally) before launching an ERAS programme, and then carefully managed throughout
Berthelsen, C.B. and Frederiksen, K. (2017). Orchestrating care through the fast track perspective: A qualitative content analysis of the provision of individualised nursing care in orthopaedic fast-track programmes. International Journal of Orthopaedic and Trauma Nursing 24: 40-49	• Includes semi-structured interviews with orthopaedic nursing staff<br>• Fast-track programmes for orthopaedic surgery (hip and knee replacement): study conducted in Denmark<br>• Focus on identifying and individual care needs and legitimising these within a fast-track programme, in order to legitimise them within fast-track nursing practices<br>• Impact of having to compromise nursing care in order to comply with the fast-track programme<br>• Conclusion: nurses felt they had to compromise their nursing care and ethics in order to comply with the fast-track programme and implement the standardised care that it recommends<br>•

One study was conducted in Canada and the US (n = 1). The sample sizes ranged from 8 to 63. The studies focus on the implementation and delivery of ERAS across a variety of clinical contexts: four on colorectal surgery[19-22], one on gastrointestinal surgery[23], one on abdominal hysterectomy[24], and two on orthopaedics[25]. Participants included in the studies were a wide range of Multidisciplinary Team and Allied Health Professional staff, and therefore incorporate a diverse range of clinical and professional perspectives. These include registrars, consultants, surgeons, anaesthetists, doctors, nurses and physiotherapists, as well as nursing managers, ERAS coordinators, care coordinators and service improvement coordinators[21]. Participants in one study were recruited specifically because of their role as local ERAS champions[20]. Individual semi-structured interviews were used for data collection in all eight studies. Two studies conducted focus groups as well as interviews[24], and one also collected and analysed memos and reflective journals completed by participants[23].

For peer review only - http://bmjopen.bmj.com/site/about/guidelines.xhtml

Analysis yielded five themes which are shown in Table 1: communication and collaboration, resistance to change, role and significance of protocol-based care, knowledge and expectations, and temporality. This enabled us to identify the key elements of health professionals' experiences of and perspectives on participation in an ERAS pathway. The themes are described in turn.

Theme 1: Communication and collaboration

Findings from all of the studies emphasised that the successful integration of ERAS practices depends upon effective multidisciplinary team (MDT) communication, and a shared willingness to collaborate. Where this worked well, comprehensive education for staff and patients about ERAS, as well as clear and effective dissemination of knowledge and information were felt to be contributing factors. The high turnover of MDT staff was cited as presenting a challenge to this process, and it was suggested that providing a 'thorough introduction'[24] about ERAS principles to new staff helped to improve matters. Good team work was also seen to be crucial[22], since this helped to foster an environment in which discipline or intervention specific concerns[19], and issues relating to staff and practice[21] could be addressed. Strong team communication was also seen as a means of mitigating staff confusion about ERAS[21]: specific areas identified as requiring improvement were communications between nurses and surgeons[19], dialogue between staff and patients, in which the compressed and information-filled approach of ERAS can prove especially challenging[24]. Having a small clinical community and a close-knit team was recognised as creating a good basis for effective organisational interactions[22].

Staff also drew attention to the challenges of coordinating the various aspects of the ERAS programme, and maintaining a good collaborative approach to this within the MDT[23]: indeed, there were concerns that a lack of coordination across different clinical departments served to

For peer review only - http://bmjopen.bmj.com/site/about/guidelines.xhtml

jeopardise ongoing consistency of practice[22], and it was felt that the provision of feedback and audits to hospital stakeholders[20] was a valuable communicative resource in this respect.

For staff working as champions, building good relationships in and across participating ERAS centres was essential for the successful integration of the programme. They recognised that such relationships served to encourage communication about - and, thereby, establish better shared understandings of current practices on the ground[20], and raise awareness about ERAS guidelines[22] by making sure that everyone's onboard. It was felt that ERAS programmes were most effectively introduced using a bottom up, as opposed to a top down approach[20]. Champions indicated that staff were more likely to engage positively with the integration of ERAS practices where they are able to be involved in co-creating them from the ground up, since this collaborative endeavour helped to foster a collective sense of responsibility[20].

Theme 2: Resistance to change

Data from the studies included in this review highlighted how *resistance to change* amongst staff had presented a major challenge to the implementation of ERAS at both collective and individual levels. It was noted, for instance, that introducing and implementing the programme requires a culture change[19,20] for staff, which they expect to find big and dramatic[23]. Concerns about the unfamiliarity of new working practices can lead to negative attitudes and a reluctance to engage with ERAS guidelines[23], whilst a fundamental dislike of change more widely also provokes disinclination[19,22]. The scope and intensity of the resistance described here is also motivated by staff age and experience[21]. Newer nurses, for instance, found it easier to adjust to the programme and tended to do so more quickly that those who were seen to be stuck in old ways[23].

Appointing a "champion" was recognised as having been extremely helpful in terms of encouraging positive attitudes and effective collaboration when implementing ERAS programmes[19,20,26]. Staff taking up this, or a similar, role were appointed from a range of MDT disciplines, and included a

For peer review only - http://bmjopen.bmj.com/site/about/guidelines.xhtml

ward based designated ERAS nurse[23] and an ERAS coordinator[21]. From the perspective of the champions themselves, meanwhile, resistance was conceptualised less broadly and in more precise terms: attributing this, for instance, to a lack of agreement about specific interventions rather than wider processes[20]. They also felt that even where MDTs were, on the whole, easily accepting of ERAS guidelines, there could still be individual level resistance[20] from some staff.

Theme 3: Role and significance of protocol based care

Staff recognised that working to evidence based protocol based guidelines can in principle be helpful, because it ‘’provides a framework to optimise patient flow by examining what should be done, when, and by whom, thereby reducing delays for patients’’[23: p.30] standardising practices, reducing variations in treatment, and thereby ostensibly improving the quality of patient care. In practice, however, there were mixed feelings amongst MDT staff as to whether or not this was the case in relation to delivering ERAS interventions. Surgeons felt that these were easily implementable as long as they were based on best evidence and incorporated in standardised order sets[19], whilst anaesthesiologists acknowledged that although they were not currently following a standardised protocol, they were open to the idea of implementing standardised guidelines[19]. There was also agreement amongst MDT staff that the implementation of the ERAS programme would provide consistency across working practices[22].

The studies highlighted several challenges of “fittingness” in relation to ERAS programmes, emphasising the relevance of institutional, organisational and patient factors. Champions noted that ERAS pathways are not definitively prescriptive, and that this leads to variability in how they ultimately becomes integrated into and operationalised within a site’s existing clinical systems[20]. One study found that needing to modify or deviate from ERAS protocols could create confusion for staff[21]. Difficulties in fitting high numbers of patients into the timescales recommended for length of hospital stay under ERAS were also cited as a challenge. Nursing staff seemed to experience the

For peer review only - http://bmjopen.bmj.com/site/about/guidelines.xhtml

greatest impact of these particular challenges on their day to day work, in which they were faced with the reality that some patients do not and cannot comply with ERAS requirements and do not "fit" standard care trajectories, because they are too frail and old, or have very high levels of comorbidity, and are simply too unwell[21,25]. Such issues presented ethical as well as logistical difficulties for nursing staff. Some described feeling highly conflicted about the tensions they experienced in striving to achieve the standardised care targets of ERAS protocols whilst also upholding their ideals of nursing practice[25]. They felt that they were having to make compromises in their work, and experienced this as a struggle. Particular concerns were raised about the detrimental impact that this was having upon nurses' capacity for providing adequately individualised care for patients[25], and the notion of having one protocol for all[23] was felt to be unsatisfactory. Nursing staff felt that the absence of clear guidance about when and how to default or deviate from ERAS protocols led them to be overly cautious in their work, and they indicated that better defined and more precise inclusion criteria about which patients to drive through recovery would be helpful[23].

Theme 4: Knowledge and expectations

Staff recognised that a good knowledge and understanding of ERAS is crucial if it is to be successfully implemented, although the scope of this requirement transcends the procedural details and pragmatic instructions provided by ERAS protocols themselves. Rather, it was important for staff to have a good grasp of its wider aims and objectives[23], and to believe in the value and (potentially) positive impact of the intervention[20,23]. Three of the studies found that, on the whole, staff did feel positive about and favourable towards the implementation of ERAS[19,20,22], and one study showed that although staff were sceptical about it prior to implementation, they felt more positive having seen how well ERAS worked in practice[24]. In all the studies, however, staff acknowledged that considerable challenges still exist and that these will need to be overcome. The nature of such concerns varied for staff, depending upon their own MDT specialty, since this had impact upon the

For peer review only - http://bmjopen.bmj.com/site/about/guidelines.xhtml

way in which they engaged with ERAS practices in their everyday work. Nurses, in particular, described feeling cautious and sceptical about implementing ERAS because of a lack of confidence, indecision, and anxieties about being challenged by other members of the MDT during ward rounds. They were also worried about any potentially adverse consequences for patients of progressing their recovery in accordance with ERAS[23]. Tacit knowledge was also understood to be important for nurses for their role in implementing ERAS: this helped them to take a common sense[23] approach to the process, especially in terms of knowing when it was appropriate to deviate from ERAS guidelines[23,25].

Setting and effectively managing expectations was a key concern for health professionals in helping them to build shared understandings around ERAS, and to understand their own individual tasks and responsibilities. The expectations of both professionals and patients (and negotiations of the two) were relevant here. Staff felt that they themselves benefitted from setting clear patient expectations[19], and were also keenly aware of some of the complex difficulties in collective understandings of what was expected from whom, when, and in which ways across the MDT, where various parties ''made an effort to fulfil the other's expectations in the situation, but from different perspectives and different understandings of the same situation''[24: p.420].

Pearsall et al.[19] note that staff expectations - of self and others - differ across the MDT and, importantly, explore how these are linked to (sometimes incorrect) assumptions made by some staff about the knowledge and expectations of their colleagues, creating uncertainty and ambivalence around ERAS implementation. For instance, where nurses anticipated that some surgeons might resist ERAS recommendations, surgeons thought that nursing culture and lack of nursing time would present a problem. Anaesthetists, meanwhile, were concerned that patients would not understand ERAS guidelines and procedures, and assumed that it would be very difficult to amend existing and well-established nursing culture and surgeon behaviours. The surgeons themselves were

For peer review only - http://bmjopen.bmj.com/site/about/guidelines.xhtml

For peer review only

unconvinced as to whether changes made in accordance with ERAS would make any difference to patients' experiences of the surgical pathway.

Staff acknowledged that their expectations about ERAS timeframes should be realistic[23], that is, accepting of the reality that some patients would be unable to achieve recovery according to the goals prescribed in the protocol. Whilst some nurses conceptualised such non-achievement as a failure of the [ERAS] programme[23], however, others saw the patients themselves as being responsible for this, on account of them being unprepared for a short hospital stay or early mobilisation, and feeling disproportionately anxious about the process[25]. Staff recognised the extent to which good pre-operative education is helpful for patients, but noted that they nevertheless have to deal with problems arising where patients have unrealistic expectations, forget important information, or simply will not comply with ERAS instructions[21]. It was also felt that some patients might be unable to understand the information and instructions that they received, creating difficulties for MDT staff[22].

Theme 5: Temporality

Temporality is highlighted as being important in findings from the studies: the successful embedding of ERAS is described, for instance, as a process that requires a slow but steady approach, becoming normalised over time, until it is established as the standard of care [so that] there's nothing to talk about[20]. The attitudes of staff towards ERAS programmes also changed over time, typically becoming more favourable as the integration continued[24], and nursing staff were keen to reflect on this, recognising how long it had taken them, to see ERAS practices as routine and second nature[23]. Some of the primary challenges identified to the effective ongoing integration of ERAS, meanwhile, were factors relating to a perceived lack of the resources (within and external to local sites) required to maintain the programme over time. These included a lack of adequate finances, limited space, a shortage of equipment, and too few nursing staff[22], as well as poor administrative

For peer review only - http://bmjopen.bmj.com/site/about/guidelines.xhtml

support for the programme[20]. Insufficient community support for discharge procedures and arrangements was also cited as a hindrance to successful embedding of ERAS.

DISCUSSION

Our meta-synthesis of qualitative studies produced five themes, which reflect key issues, concerns and areas of importance identified by health professionals in relation to their experiences of delivering ERAS pathways. These themes were communication and collaboration, resistance to change, role and significance of protocol-based care, knowledge and expectations, and temporality. Staff emphasised that there must be effective MDT collaboration and communication, if ERAS practices are to be successfully implemented and integrated. This included providing a thorough education to staff and patients about ERAS, and ensuring that information and knowledge about it was clearly and consistently disseminated across the MDT. The coordination of ERAS approaches was acknowledged to be challenging, and the appointment of a designated ERAS champion was experienced as being helpful in this respect.

The value of evidence based protocol based guidelines was described as useful means of helping to improve patient care by bringing about a standardisation of practices and a reduction in variations in treatment, but staff were ambivalent about the extent to which ERAS created such consistencies in practice. Concerns were raised about the necessity of modifying or deviating from ERAS guidelines, where these did not “fit” with local site systems or with the care requirements of individual patients. A need for more precise information about how best to do this was identified.

A comprehensive knowledge and understanding of ERAS was cited as being essential to its successful implementation: in terms of both procedural detail and the broader aims and objectives that underpin the intervention itself. Staff were concerned about the impact of ERAS upon their own everyday working practices, and in relation to their own particular MDT speciality. Staff expectations

For peer review only - http://bmjopen.bmj.com/site/about/guidelines.xhtml

about ERAS varied across MDT disciplines, and the need to set and manage these effectively was prioritised. The importance of establishing 'realistic' expectations was emphasised for staff and also the patients for whom they care.

The implementation and embedding of ERAS was understood to require complex processes of adjustment, acceptance and engagement for staff, constituting a process that evolves gradually over time. Staff attitudes towards ERAS were also subject to temporal change, and tended to become increasingly favourable via reflections upon how well the new and or amended practices were working, and the ways in which they became 'normalised'.

Given that ERAS seeks to improve patients' outcomes through consistency in care, findings from our review highlight that, whilst health professionals are confident that ERAS pathways have the potential to achieve this, some key improvements are needed. The findings of this review build upon existing knowledge about ERAS by showing that the pathway is implemented disparately across different settings, according to local contexts and circumstances[4], and that the provision of better information and education to staff and patients can achieve better consistency. Our review also finds that health professionals cite resistance to change amongst staff as a hindrance to the effective implementation of ERAS[6], and that appointing a dedicated Enhanced Recovery "champion" is felt to mitigate this[7]. Our findings demonstrate that effective collaboration and communication amongst staff – and between staff and patients – helps to improve the effectiveness of ERAS[5]. This review also observes that staff use different strategies of discursive framing to describe their experiences of implementing and engaging with ERAS practices, which indicate that temporality is an important factor in this respect. These include accounts of adapting to, adjusting to, and coming around to the programme[23], adopting the pathway[19], being accepting of it[20], and compliance with ERAS guidelines[21]. From a discursive psychological perspective – which focuses upon social

For peer review only - http://bmjopen.bmj.com/site/about/guidelines.xhtml
For peer review only

construction through language[27], these framings illustrate how the implementation of ERAS is produced through talk[28] as a gradual process which evolves over time.

We conducted the systematic review in a manner that was designed to capture as many studies as possible by using keywords that were identified and refined from existing literature. To enhance rigour in study selection the included studies were all appraised by the two authors. This process acted as a screening process that allowed us to exclude three studies and retain eight as well as appraise whether the included studies met the CASP criteria sufficiently. Assessment using the CASP criteria can be conducted in a variety of ways and our process enabled us to define all studies to be of sufficient quality. To improve reporting quality of this review, we have adhered to the ENTREQ guidance on the reporting of qualitative syntheses[29]. The reflexive approach of the authors in the selection process sought to minimise researcher bias.

One of the strengths of this review is that it includes a range of different studies, and therefore incorporates a variety of populations and geographical contexts. Further strengths are the diversity of methodological approaches used in the studies, and the different clinical contexts and local environments of the included studies. This provides a richness of perspectives. Additionally, there are few existing systematic reviews of qualitative studies on staff experiences of ERAS. This paper therefore makes a valuable contribution to the field of literature. A limitation of this review is the small number of included studies, which reduce the level of transferability of our findings. Another limitation is that there are no ethnographic studies included in our review and, as such, it does not benefit from the use of observational data on the implementation of ERAS. We suggest that future research could build upon existing knowledge of and understanding about staff perspectives of ERAS by taking an ethnographic approach. We also note that ERAS pathways are now being implemented in elective orthopaedic surgery, and suggest that this is a valuable area for future study.

For peer review only

CONCLUSION

We reviewed and synthesised qualitative studies that explore health professionals' experiences of and perspectives on the enhanced recovery after surgery (ERAS) pathway. Findings indicate that, whilst staff generally feel positive about the implementation of ERAS, they acknowledge that the process is complex and challenging. Many of the challenges identified, such as resistance to change and lack of confidence can however be mitigated by ensuring that MDTs understand ERAS principles and guidelines, and that they communicate well with one another and with patients. Other challenges, such as a lack of local resources and high rates of comorbidity amongst patients are perhaps more challenging to address. We suggest that the provision of comprehensive, coherent and locally relevant information to health professionals would help to improve the implementation and delivery of ERAS pathways. Identifying and recruiting an ERAS champion is also recommended as means of improving the effectiveness of the pathway.

A: CONTRIBUTORSHIP STATEMENT

The authors of this article are Dr Rachel Cohen (RC) and Professor Rachael Gooberman-Hill (RGH). Both authors made substantial contributions to the conception and the design of the systematic review. Literature searches were conducted by RC, and RGH carried out the CASP screening. Both RC and RGH contributed to the extraction, analysis and interpretation of data from the papers included in the review. RC and RGH worked on drafts of the review, made revisions and agreed on a final version for publication. Both RC and RGH agree to be accountable for all aspects of the work in ensuring that questions related to the accuracy or integrity of any part of the work are appropriately investigated and resolved.

For peer review only - http://bmjopen.bmj.com/site/about/guidelines.xhtml

Support for the study was received from the Oxford NIHR Biomedical Research Centre, Nuffield Orthopaedic Centre, University of Oxford. The views and opinions expressed therein are those of the authors and do not necessarily reflect those of the HS&DR Programme, NIHR, NHS or the Department of Health. We thank the wider team for the study, including Professor Andrew Judge as Principal Investigator. We also thank Christine Hobson for her work in support of our research.

B: COMPETING INTERESTS

The author and co-author have no competing interests to declare

C: FUNDING

This review forms part of the dissemination strategy for the Atlas (Ethnographic study of care pathways for hip and knee replacement) project, which is funded by the National Institute for Health Research Health Services and Delivery Research Programme (project number 14/46/02).

D: DATA SHARING STATEMENT

Data for the study may be made available from University of Bristol's research data repository under a controlled access arrangement. Requests for access will be referred to the University’s data access committee before data can be shared under a data sharing agreement. As such, anonymous data from the study may be seen and used by other researchers, for ethically approved research projects, on the understanding that confidentiality will be maintained. Release of the data will be at the discretion of the data access committee (data custodian).

For peer review only - http://bmjopen.bmj.com/site/about/guidelines.xhtml

REFERENCES

1. Kehlet H. Fast-track colorectal surgery. *The Lancet* 2008;371:791-3.

2. Department of Health. Enhanced Recovery Partnership Programme Project Report. March 2011.Available from: https://www.gov.uk/government/publications/enhanced-recovery-partnership-programme (accessed 1 Feb 2017).

3. Campbell NC, Murray E, Darbyshire J, *et al*. Designing and evaluating complex interventions to improve health care. *BMJ* 2007;334:455-9.

4. Craig P, Dieppe P, Macintyre S, *et al.* Developing and Evaluating Complex Interventions: The New Medical Research Council Guidance. *BMJ* 2008;337:979-83.

5. Waring J, Marshall F, Bishop S, *et al*. An ethnographic study of knowledge sharing across the boundaries between care processes, services and organisations: the contributions to 'safe' hospital discharge. *Health Serv Deliv Res* 2014:2;1-160.

6. Moore G, Audrey S, Barker M, *et al*. Process evaluation in complex public health intervention studies: the need for guidance. *J Epidemiol Community Health* 2014;68:101-2.

7. Paton F, Chambers D, Wilson P, *et al.* Effectiveness and implementation of enhanced recovery after surgery programmes: a rapid evidence synthesis. *BMJ Open* 2014;4:e005015. doi:10.1136/bmjopen-2014-005015.

8. Bernard H, Foss M. The impact of the enhanced recovery after surgery (ERAS) programme on community nursing. *Br J Community Nurs* 2014;19:184-8.

For peer review only - http://bmjopen.bmj.com/site/about/guidelines.xhtml

9. Blazeby JM, Soulsby M, Winstone K, *et al*. A qualitative evaluation of patients' experiences of an enhanced recovery programme for colorectal cancer. *Colorectal Dis* 2010;12:e236-42.

10. Hunt GR, Hall GM, Murthy BV, *et al*. Early Discharge Following hip Arthroplasty: Patients' acceptance masks doubts and concerns. *Health Expect* 2009;12:130-7.

11. Husted H, Holm G. Fast track in total hip and knee arthroplasty - experiences from Hvidovre University Hospital, Denmark. *Injury* 2006;37(Suppl 5):S31-S35.

12. Norlyk A. The experience of fast track postoperative rehabilitation regimen: the patients' perspective. *Klinisk Sygepleje* 2008;22:53-63.

13. Norlyk A, Harder I. After colonic surgery: The lived experience of participating in a fast-track programme. *Int J Qual Stud on Health Well-being* 2009;4:170-180.

14. Sibbern T, Bull Sellevold V, Steindal SA, *et al*. Patients' Experiences of Enhanced Recovery after Surgery: a systematic review of qualitative studies. *J Clin Nurs* 2017;26:1172-188.

15. Critical Skills Appraisal Programme. *CASP Systematic Review Checklist*. Available from: http://www.casp-uk.net/checklists (accessed 1 Feb 2016).

16. Polit DF, Beck CT. Nursing Research: Generating and Assessing Evidence for Nursing Practice. Philadelphia: Wolters Kluwer/Lippincott Williams and Wilkins; 2008.

17. Graneheim UH, Lundman B. Qualitative content analysis in nursing research: concepts, procedures and measures to achieve trustworthiness. *Nurse Educ Today* 2004;24:105–12.

For peer review only - http://bmjopen.bmj.com/site/about/guidelines.xhtml

18. Uhrenfeldt L, Aagaard H, Hall EO, *et al.* A qualitative meta-synthesis of patients' experiences of intra- and inter-hospital transitions. *J Adv Nurs* 2013;69:1678–90.

19. Pearsall EA, Meghiji A, Pitzul KB, *et al*. A Qualitative Study to Understand the Barriers and Enablers in Implementing an Enhanced Recovery after Surgery Program. *Ann Surg* 2015;261:92-6.

20. Gotlib Conn L, McKenzie M, Pearsall EA, *et al*. Successful implementation of an enhanced recovery after surgery programme for elective colorectal surgery: a process evaluation of champions' experiences. *Implement Sci* 2015;10:99.

21. Lyon A. Solomon MJ, Harrison JD. A Qualitative Study Assessing the Barriers to Implementation of Enhanced Recovery after Surgery. *World J Surg* 2014;38:1374-80.

22. Alawadi ZM, Leal I, Phatak UR, *et al*. Facilitators and barriers of implementing enhanced recovery in colorectal surgery at a safety net hospital: A provider and patient perspective. *Surgery* 2016;159:700-12.

23. Jeff A, Taylor C. Ward nurses' experiences of enhanced recovery after surgery: a grounded theory approach. *Gastrointestinal Nursing* 2014;12:23-31.

24. Wagner L, Carlslund AM, Moller C, *et al.* Patient and staff (doctors and nurses) experiences of abdominal hysterectomy in accelerated recovery programme: A qualitative study. *Dan Med Bull* 2004;51:418-21.

For peer review only - http://bmjopen.bmj.com/site/about/guidelines.xhtml

25. Berthelsen CB, Frederiksen K. Orchestrating care through the fast track perspective: A qualitative content analysis of the provision of individualised nursing care in orthopaedic fast-track programmes. *Int J Orthop Trauma Nurs* 2017;24:40-9.

26. Sjetne IS, Krogstad U, Odegard, S, *et al*. Improving quality by introducing enhanced recovery after surgery in a gynaecological department: consequences for ward nursing practice. Qual Saf Health Care; 2009:18;236-40.

27. Wiggins S, Potter J. Discursive Psychology. In: Willig C, Hollway SR, eds. *Handbook of Qualitative Research*. London:Sage 2008:72-89.

28. Potter J, Wetherell M. *Discourse and Social Psychology: Beyond Attitudes and Behaviour.* London: Sage, 1987.

29. Tong A, Flemming K, McInnes *et al*. Enhancing transparency in reporting the synthesis of qualitative research: ENTREQ. *BMC Med Res Methodol* 2012;12:181.

For peer review only

For peer review only - http://bmjopen.bmj.com/site/about/guidelines.xhtml

PRISMA 2009 Flow Diagram
Staff experiences of Enhanced Recovery after Surgery – Systematic Review of Qualitative Studies

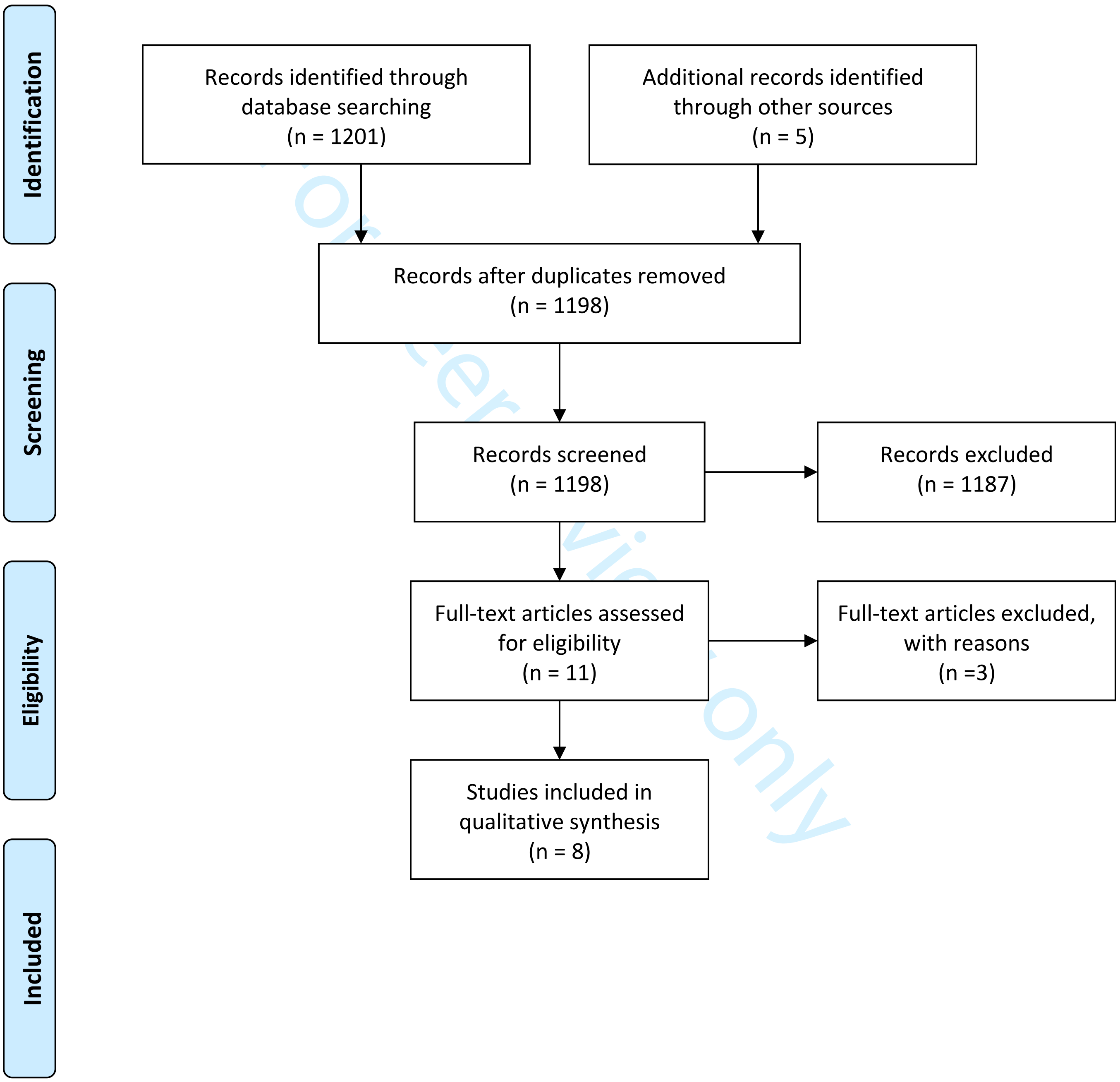

From: Moher D, Liberati A, Tetzlaff J, Altman DG, The PRISMA Group (2009). *P*referred *R*eporting *I*tems for *S*ystematic Reviews and *M*eta-*A*nalyses: The PRISMA Statement. PLoS Med 6(7): e1000097. doi:10.1371/journal.pmed1000097

For more information, visit www.prisma-statement.org.

For peer review only - http://bmjopen.bmj.com/site/about/guidelines.xhtml

PRISMA 2009 Checklist

Section/topic	#	Checklist item	Reported on page #
**TITLE**			
Title	1	Identify the report as a systematic review, meta-analysis, or both.	1
**ABSTRACT**			
Structured summary	2	Provide a structured summary including, as applicable: background; objectives; data sources; study eligibility criteria, participants, and interventions; study appraisal and synthesis methods; results; limitations; conclusions and implications of key findings; systematic review registration number.	2
**INTRODUCTION**			
Rationale	3	Describe the rationale for the review in the context of what is already known.	4
Objectives	4	Provide an explicit statement of questions being addressed with reference to participants, interventions, comparisons, outcomes, and study design (PICOS).	5
**METHODS**			
Protocol and registration	5	Indicate if a review protocol exists, if and where it can be accessed (e.g., Web address), and, if available, provide registration information including registration number.	5
Eligibility criteria	6	Specify study characteristics (e.g., PICOS, length of follow-up) and report characteristics (e.g., years considered, language, publication status) used as criteria for eligibility, giving rationale.	6
Information sources	7	Describe all information sources (e.g., databases with dates of coverage, contact with study authors to identify additional studies) in the search and date last searched.	5,6
Search	8	Present full electronic search strategy for at least one database, including any limits used, such that it could be repeated.	5,6
Study selection	9	State the process for selecting studies (i.e., screening, eligibility, included in systematic review, and, if applicable, included in the meta-analysis).	5,6,7
Data collection process	10	Describe method of data extraction from reports (e.g., piloted forms, independently, in duplicate) and any processes for obtaining and confirming data from investigators.	6-9
Data items	11	List and define all variables for which data were sought (e.g., PICOS, funding sources) and any assumptions and simplifications made.	6-9
Risk of bias in individual studies	12	Describe methods used for assessing risk of bias of individual studies (including specification of whether this was done at the study or outcome level), and how this information is to be used in any data synthesis.	6-9
Summary measures	13	State the principal summary measures (e.g., risk ratio, difference in means).	N/A
Synthesis of results	14	Describe the methods of handling data and combining results of studies, if done, including measures of consistency (e.g., I^2) for each meta-analysis.	6-9

For peer review only

For peer review only - http://bmjopen.bmj.com/site/about/guidelines.xhtml

PRISMA 2009 Checklist

Section/topic	#	Checklist item	Reported on page #
Risk of bias across studies	15	Specify any assessment of risk of bias that may affect the cumulative evidence (e.g., publication bias, selective reporting within studies).	N/A
Additional analyses	16	Describe methods of additional analyses (e.g., sensitivity or subgroup analyses, meta-regression), if done, indicating which were pre-specified.	N/A
**RESULTS**			
Study selection	17	Give numbers of studies screened, assessed for eligibility, and included in the review, with reasons for exclusions at each stage, ideally with a flow diagram.	6,7
Study characteristics	18	For each study, present characteristics for which data were extracted (e.g., study size, PICOS, follow-up period) and provide the citations.	10-12
Risk of bias within studies	19	Present data on risk of bias of each study and, if available, any outcome level assessment (see item 12).	N/A
Results of individual studies	20	For all outcomes considered (benefits or harms), present, for each study: (a) simple summary data for each intervention group (b) effect estimates and confidence intervals, ideally with a forest plot.	N/A
Synthesis of results	21	Present results of each meta-analysis done, including confidence intervals and measures of consistency.	N/A
Risk of bias across studies	22	Present results of any assessment of risk of bias across studies (see Item 15).	N/A
Additional analysis	23	Give results of additional analyses, if done (e.g., sensitivity or subgroup analyses, meta-regression [see Item 16]).	N/A
**DISCUSSION**			
Summary of evidence	24	Summarize the main findings including the strength of evidence for each main outcome; consider their relevance to key groups (e.g., healthcare providers, users, and policy makers).	19-21
Limitations	25	Discuss limitations at study and outcome level (e.g., risk of bias), and at review-level (e.g., incomplete retrieval of identified research, reporting bias).	21,22
Conclusions	26	Provide a general interpretation of the results in the context of other evidence, and implications for future research.	21,22
**FUNDING**			
Funding	27	Describe sources of funding for the systematic review and other support (e.g., supply of data); role of funders for the systematic review.	23

From: Moher D, Liberati A, Tetzlaff J, Altman DG, The PRISMA Group (2009). Preferred Reporting Items for Systematic Reviews and Meta-Analyses: The PRISMA Statement. PLoS Med 6(7): e1000097. doi:10.1371/journal.pmed1000097

For more information, visit: **www.prisma-statement.org**.

For peer review only - http://bmjopen.bmj.com/site/about/guidelines.xhtml

BMJ Open

Staff Experiences of Enhanced Recovery after Surgery – Systematic Review of Qualitative Studies

Journal:	*BMJ Open*
Manuscript ID	bmjopen-2018-022259.R1
Article Type:	Research
Date Submitted by the Author:	18-Jun-2018
Complete List of Authors:	Cohen, Rachel; University of Bristol, School of Population Health Sciences<br>Gooberman-Hill, Rachael; University of Bristol, School of Clinical Sciences
**Primary Subject Heading**:	Health services research
Secondary Subject Heading:	Qualitative research
Keywords:	Enhanced recovery, Qualitative, Joint replacement

SCHOLARONE™ Manuscripts

For peer review only

Article Title: STAFF EXPERIENCES OF ENHANCED RECOVERY AFTER SURGERY – SYSTEMATIC REVIEW OF QUALITATIVE STUDIES

Authors: [#]Dr Rachel Cohen and Professor Rachael Gooberman-Hill*

***Corresponding author:** Dr Rachel Cohen, Centre for Academic Mental Health, Department of Population Health Sciences, Bristol Medical School, University of Bristol, Bristol, BS8 2BN, UK

Corresponding author's Email address: rachel.cohen@bristol.ac.uk Tel: +44(0)117 331 4007

[#]Co-author: Professor Rachael Gooberman-Hill, Professor of Health and Anthropology, Translational Health Sciences, Bristol Medical School, University of Bristol, Learning and Research Building, Level 1, Southmead Hospital, Bristol BS10 5NB, UK

Word count: 4194

Keywords: Enhanced Recovery, qualitative, joint replacement

For peer review only

ABSTRACT

Objective: To conduct a systematic review of qualitative studies which explore health professionals' experiences of and perspectives on the enhanced recovery after surgery (ERAS) pathway.

Design: Systematic review of qualitative literature using a qualitative content analysis.

Setting: Secondary care, including a wide range of hospital settings.

Participants: Health professionals including a wide range of Multidisciplinary Team and Allied Health Professional staff to incorporate a diverse range of clinical and professional perspectives.

Results: Eight studies were included in the review, including studies in six countries and in four surgical specialties. Included studies focus on health professionals' experiences of ERAS before, during and after implementation in colorectal surgery, gastrointestinal surgery, abdominal hysterectomy, and orthopaedics. Five main themes emerged in the analysis: communication and collaboration, resistance to change, role and significance of protocol-based care, knowledge and expectations, and temporality. Professionals described the importance of effective multidisciplinary team collaboration and communication, providing thorough education to staff and patients, and appointing a dedicated champion as means to implement and integrate ERAS pathways successfully. Evidence based guidelines were thought to be useful for improvements to patient care by standardising practices and reducing treatment variations, but were thought to be too open to interpretation at local levels. Setting and managing 'realistic' expectations of staff was seen as a priority. Staff attitudes towards ERAS tend to become more favourable over time, as practices become successfully 'normalised'.

Conclusions: Staff feel positive about the implementation of ERAS, but find the process is complex and challenging. Challenges can be addressed by ensuring that multidisciplinary teams understand ERAS principles and guidelines, and communicate well with one another and with patients. Provision of comprehensive, coherent and locally relevant information to health professionals is helpful. Identifying and recruiting local ERAS champions is likely to improve the implementation and delivery of ERAS pathways.

For peer review only - http://bmjopen.bmj.com/site/about/guidelines.xhtml

ARTICLE SUMMARY

Strengths and limitations of this study

- There is a need to synthesise qualitative evidence about staff experiences of Enhanced Recovery After Surgery (ERAS) because these provide insight into implementation of ERAS
- The review includes studies with a diverse range of populations, contexts and methodological approaches
- The review includes a small number of studies, but includes studies from six countries in four surgical specialties and so the findings are likely to be transferable and of relevance to several contexts of ERAS implementation

For peer review only

For peer review only - http://bmjopen.bmj.com/site/about/guidelines.xhtml

INTRODUCTION

Enhanced recovery after surgery (ERAS) programmes were introduced and began to be implemented in the late 1990s[1], as part of an initiative towards reducing variations in patient care and improving quality standards[2]. Building upon their Danish origins, ERAS programmes have been internationally adopted, and widely implemented for major elective surgical pathways in colorectal surgery, orthopaedics, gynaecology, cardiology and urology. Depending upon the kind of diagnostic and surgical care in question, ERAS programmes are sometimes referred to using different names, including 'fast-track surgery', 'rapid recovery', 'accelerated discharge' or 'early discharge'.

The aim of ERAS pathways is to reduce length of hospital stay and lessen readmissions, minimise surgical complications, decrease morbidity, and improve cost effectiveness. Best described as a complex intervention[3,4], ERAS seeks to improve patient experiences and outcomes by focusing on key aspects of the care pathway, pre-, peri- and post operatively, as a means of reducing physiological and psychological stress. This involves the provision of better education and information for patients prior to their operations, the use of minimally invasive surgical techniques and anaesthesia, optimal pain management and early post-operative mobilisation, as well as the preparation of a discharge plan[5].

Despite their protocol-based foundations, evidence from recent studies indicates that ERAS pathways are implemented variably across different hospital settings. More information is needed about what the core active ingredients of ERAS are. We also need to know more about how these ingredients exert their effect according to local circumstances, and about how they shape (and are shaped by) the context of their implementation[4,6]. Existing literature has drawn particular attention to the factors which help, and those which hinder, the successful implementation of ERAS, identifying important barriers and facilitators to the process Barriers include resistance to change, inadequate funding, lack of support from management, high staff turnover, poor documentation,

For peer review only

For peer review only - http://bmjopen.bmj.com/site/about/guidelines.xhtml

and shortness of time, whilst facilitators included a dedicated enhanced recovery lead, effective multidisciplinary team working and ongoing education for staff and patients[7].

Patient experiences of and satisfaction with ERAS pathways have been studied using both quantitative and qualitative approaches: the latter have been especially useful in improving understandings of patient experiences and perspectives e.g.[8,9,10,11,12,13]. Sibbern et al's[14] systematic review of studies of patients' experiences provides a comprehensive discussion of existing qualitative research on this specific topic. Health professionals' satisfaction with and perspectives on ERAS, meanwhile, have typically been explored using quantitative approaches. Information about the experiences of health professionals in delivery of ERAS is needed to inform implementation and healthcare policy and practice. Such experiences are best gathered in details through qualitative research.

This article describes a systematic review of qualitative studies of health professionals' experiences of ERAS pathways. The aim of the review was to synthesise evidence of the experience of health professionals who have been involved in implementing the ERAS programme, incorporating their experiences before, during and after the programme was implemented, and of its subsequent delivery. The review aims to identify overarching themes that provide opportunities for improving implementation and practice.

METHODS

Patient and Public Involvement

This paper is a systematic review of qualitative studies. No patients were involved in the review. Our systematic review was registered on PROSPERO in 2017: the registration number is CRD42017059952. The review sought to describe the experiences and perspectives of healthcare professionals involved in delivering enhanced recovery pathways.

For peer review only - http://bmjopen.bmj.com/site/about/guidelines.xhtml

We used methods of systematic search and review and conducted a search of PsychINFO, Medline, Cinahl, and PubMed to identify relevant qualitative studies across a range of health care contexts. The searches were conducted by the first author and checked by the second author. The searches included studies published from 2000-2017, as an appropriate timeframe to capture evidence about ERAS after implementation in the late 1990s. Only studies published in the English language were included, and we included studies that explicitly stated that they used qualitative approaches. For all of the databases, the search terms used were:

> ERAS OR enhanced recovery OR fast-track OR accelerated recovery OR rapid recovery OR early discharge OR patients discharge OR enhance* recov* after surg*
>
> Staff perspective OR staff experience* OR staff perception* OR ward staff OR nurs* OR professional*
>
> Qualitative OR interview* OR ethnograph* OR observation

The reference lists of articles identified from the database search were also scrutinised for possible additional studies.

As shown in the PRISMA flowchart (supplementary file), the database searches yielded 1201 articles in total. In addition, through searching the reference lists of the included studies, we identified five further records. Eleven studies met the inclusion criteria, and were assessed for eligibility using the Critical Appraisal Skills Programme (CASP) guidance (Critical Skills Appraisal Programme 2017)[15]. The CASP checklist for qualitative research provides a means of identifying the strengths and weaknesses of research articles, assessing their usefulness and validity, and their relevance for inclusion in the review. The CASP qualitative checklist was designed as a pedagogic tool and therefore as a means of assessing whether qualitative approaches are appropriate to a research

For peer review only - http://bmjopen.bmj.com/site/about/guidelines.xhtml

For peer review only

question, the value of results, and to provide the opportunity to assess quality in a qualitative, expertise-based and discursive fashion. Therefore, we considered the eleven studies using the ten CASP questions which are: aim, methodology, design, recruitment strategy, data collection, relationship between researcher and participants, ethical issues, data analysis, findings and research value - three studies were excluded, and the remaining eight were included. Two of the three that were excluded at this stage were quantitative rather than qualitative, and one focused on rehabilitation following hip and knee arthroplasty, but not specifically on ERAS. The two authors independently conducted quality assessment and agreed that all eight articles addressed all ten CASP criteria and were of sufficient rigour and relevance for inclusion in the review.

After completion of quality assessment we conducted a qualitative meta-synthesis of the eight eligible articles. This comprised close reading and extraction of key findings using descriptive qualitative design[16], and a qualitative content analysis[17,18]. For the analysis, we focused on the manifest content of the articles, i.e. what the texts say[17]. This involved searching for the common concepts and themes[18] addressed in the articles regarding health professionals' experiences of and perspectives on ERAS. Supporting quotes were also gathered. This enabled us to develop meaning units within the themes, with the meaning units extracted from the findings of the studies. Meaning units refer to the main considerations in relation to each theme that were raised by staff about their experiences of implementing and delivering ERAS programmes. These were then condensed into content related categories, which the authors discussed and agreed upon. Content related categories refer to the suggested techniques for addressing and responding to these considerations. We then synthesised the chosen categories into themes as shown in Table 1.

For peer review only - http://bmjopen.bmj.com/site/about/guidelines.xhtml

TABLE 1

Theme	Meaning Unit	Content related category
Collaboration and communication	• Staff find the information-rich nature of ERAS confusing. Many staff feel that they do not understand it well enough and/or that they have not received sufficiently clear or consistent information or training<br>• Information about ERAS is not always disseminated between staff – and between staff and patients – in a coherent and consistent way.<br>• Collaborative MDT work is hindered by high staff turnover and a lack of coordination across different departments	• Providing staff and their patients with a comprehensive education about and introduction to ERAS improves understanding and helps to mitigate confusion<br>• Strong team communications help to ensure the effective dissemination of information<br>• Building good relationships within the MDT helps to encourage dialogue between staff, and to improve their willingness and ability to collaborate. The appointment of a dedicated ERAS 'champion' improves staff engagement and collaborative working
Resistance to change	• Staff are reluctant to implement or engage with new and unfamiliar working practices. Some staff – especially those who are older, or more well established in their role – tend to dislike change more generally and are disinclined to engage with ERAS.	• Appointing and ERAS champion helps to encourage more positive attitudes amongst staff
Role and significance of protocol based care	• Staff recognise the usefulness of evidence-based protocol guidelines as a means of reducing variations and standardising practice, but have mixed feelings about whether ERAS facilitates this well.<br>• ERAS is not definitively prescriptive, and therefore allows for too much variability in local implementation.<br>• Some staff feel conflicted about having to compromise their capacity for and confidence in providing	• The incorporation of standardised order sets and basing ERAS practices on best evidence increases staff willingness to implement it as a complex intervention<br>• Having a local ERAS champion helps to improve consistency in implementing and operationalising the pathway into existing systems at local sites<br>• Clearer guidance about when

For peer review only - http://bmjopen.bmj.com/site/about/guidelines.xhtml

	individualised care for patients.	it is acceptable to deviate from ERAS protocols would improve staff confidence.
Knowledge and expectations	• Staff feel that they need a broader knowledge and understanding of ERAS, i.e. beyond protocol guidelines.<br>• Staff are sceptical about the usefulness and value of ERAS prior to its implementation.<br>• Managing the expectations of staff and patients is recognised as being crucial to the successful implementation of ERAS. Differing professional perspectives, which are sometimes based on incorrect assumptions, can create ambivalence and uncertainty amongst staff. Staff use tacit knowledge and a "common sense" approach to overcome this.	• Belief in the value and potential positive impact of ERAS improves the willingness of staff to engage with the pathway and its guidelines.<br>• Staff feel more positive about and favourable towards ERAS when they have seen it work successfully in practice.<br>• Setting clear and realistic expectations about ERAS helps to improve staff and patient experiences of the pathway.
Temporality	• The successful implementation and embedding of ERAS is a gradual process.	• ERAS becomes "normalised" over time, and staff attitudes towards the pathway tend to become more positive. There are ongoing challenges with a lack of available resources (financial and administrative) that are necessary to successfully maintain ERAS over time.

For peer review only

For peer review only - http://bmjopen.bmj.com/site/about/guidelines.xhtml

For peer review only

For peer review only - http://bmjopen.bmj.com/site/about/guidelines.xhtml

FINDINGS

The eight studies included were conducted in the UK (n = 1), US (n = 1), Canada (n = 2), Denmark (n = 2), Norway (n=1) and Australia (n = 1) (Table 2).

TABLE 2

Study	Study design	Surgical population	Methodology and methods	Number and type of participants	Country	Key findings
Alawadi et al	Qualitative study to assess the perceived barriers and facilitators before ERAS adoption.	Colorectal surgery	Qualitative interviews with MDT staff and patients. Content analysis	8 anaesthesiologists, 5 surgeons, 6 nurses, and 18 patients	US	Conclusion: 'Although limited hospital resources are perceived as a barrier to ERAS implementation… there is strong support for such pathways and multiple factors were identified that may facilitate change' (2016: 700)
Sjetne et al	Pre-postintervention	Gynaecological	Questionnaires and	34, 33 and 32 nurses	Norway	Conclusion:

For peer review only - http://bmjopen.bmj.com/site/about/guidelines.xhtml

For peer review only

	prospective design, to monitor changes in workload and work environment of ward nursing staff when ERAS was introduced	surgery	qualitative interviews. Quantitative data analysed using SAS (t tests and differences in means), qualitative data used to elaborate the topics studied	returned questionnaires in phases 1,2, and 3 respectively (100% survey response rate)<br>9 interviews with 4 different nurses		'expected clinical gains achieved by introducing ERAS are achieved without compromising the work environment of ward nurses' (2009: 239)
Pearsall et al	Qualitative study to understand barriers and enablers in perioperative implementation of ERAS	Colorectal surgery	Qualitative semi structured interviews. Thematic analysis	19 general surgeons, 18 anaesthesiologists, 18 nurses	Canada	Conclusion: 'participants supported the need for implementation of an ERAS program… [but] felt there remained major barriers to [its] successful implementation' (2015: 96)
Wagner et al	Exploratory and descriptive qualitative study to gather knowledge about staff and patient experiences of the Accelerated Recovery Programme (ARP)	Abdominal hysterectomy	Qualitative individual interviews and focus groups with staff, observation of and interviews with patients. Thematic analysis.	Observation of 17 patients, 10 of whom were interviewed twice<br>Interviews with 15 staff, who all participated in focus groups	Denmark	Conclusion: patients underwent ARP without significant problems, but identified a need for greater psychological support. Staff data showed a positive

For peer review only - http://bmjopen.bmj.com/site/about/guidelines.xhtml

						change in opinion and an understanding of ARP. Recommendations made for better information to be provided to staff and patients, in consultation rooms and outpatient clinics
Jeff et al	To explore and describe ward nurses' experience of ERAS in the postoperative phase	Gastrointestinal surgery	Semi structured interviews and documentary evidence (memos and reflective journals). Thematic analysis.	Interviews with 8 (of a possible 30) nurses	UK	Conclusion: 'the central difficulty experienced by nurses was trying to adapt the protocol to the demands of patient care delivery within the constraints of their role and organisational culture' (2014: 31)
Gotlib Conn et al	Process evaluation of ERAS champions' experiences. To understand enablers and barriers to the successful	Colorectal surgery	Qualitative semi structured interviews. Normalisation Process Theory framework analysis.	5 surgeons, 14 anaesthesiologists, 15 nurses, and 14 project coordinators	Canada	Conclusion: successful implementation of ERAS is achieved by a 'complex series of cognitive and social

For peer review only - http://bmjopen.bmj.com/site/about/guidelines.xhtml

	implementation of ERAS					processes… [the study demonstrates the importance of] champion coherence, external and internal relationship building, and the strategic management of a project's organisation-level visibility' (2015: 1)
Lyon et al	Qualitative study to assess barriers to ERAS implementation, conducted at post-operative stage	Colorectal surgery	Qualitative semi-structured interviews. Grounded theory analysis.	18 interviews with MDT staff	Australia	Conclusion: there are four key areas that present barriers to successful ERAS implementation: (i) patient-related factors, (ii) staff-related factors, (iii) practice-related issues and (iv) resources. For ERAS to be implemented successfully and function efficiently with high levels of

For peer review only - http://bmjopen.bmj.com/site/about/guidelines.xhtml

						compliance, these key areas need to be addressed (ideally) before launching an ERAS programme, and then carefully managed throughout
Berthelsen et al	Qualitative study to illuminate orthopaedic nurses' perceptions and experiences of providing individual nursing care for older patients in standardised fast-track programmes	Orthopaedic surgery (hip and knee replacement)	Semi-structured interviews. Manifest and latent content analysis	10 interviews with orthopaedic nurses	Denmark	Conclusion: nurses felt they had to compromise their nursing care and ethics in order to comply with the fast-track programme and implement the standardised care that it recommends

For peer review only

For peer review only - http://bmjopen.bmj.com/site/about/guidelines.xhtml

The sample sizes ranged from 8 to 63. The studies focus on the implementation and delivery of ERAS across a variety of clinical contexts: four on colorectal surgery[19,20,21,22], one on gastrointestinal surgery[23], one on abdominal hysterectomy[24], and two on orthopaedics[25]. Participants included in the studies were a wide range of Multidisciplinary Team and Allied Health Professional staff, and therefore incorporate a diverse range of clinical and professional perspectives. These include registrars, consultants, surgeons, anaesthetists, doctors, nurses and physiotherapists, as well as nursing managers, ERAS coordinators, care coordinators and service improvement coordinators[21]. Participants in one study were recruited specifically because of their role as local ERAS champions[20]. Individual semi-structured interviews were used for data collection in all eight studies. Two studies conducted focus groups as well as interviews[24], and one also collected and analysed memos and reflective journals completed by participants[23]. The different methodologies used in the included studies emphasise the usefulness of this review in drawing together a range of perspectives on staff experiences of implementing ERAS programmes. Despite their different contexts and surgical populations, the findings from the included studies were largely consistent with one another.

Analysis yielded five themes which are shown in Table 1: communication and collaboration, resistance to change, role and significance of protocol-based care, knowledge and expectations, and temporality. The themes identify the key elements of health professionals' experiences of and perspectives on participation in an ERAS pathway. Each theme is described in turn.

Theme 1: Communication and collaboration

Findings from all of the studies emphasised that the successful integration of ERAS practices depends upon effective multidisciplinary team (MDT) communication, and a shared willingness to

For peer review only - http://bmjopen.bmj.com/site/about/guidelines.xhtml

collaborate. Where this worked well, comprehensive education for staff and patients about ERAS, as well as clear and effective dissemination of knowledge and information were felt to be contributing factors. The high turnover of MDT staff was cited as presenting a challenge to this process, and it was suggested that providing a 'thorough introduction'[24] about ERAS principles to new staff helped to improve matters. Good team work was also seen to be crucial[22], since this helped to foster an environment in which discipline or intervention specific concerns[19], and issues relating to staff and practice[21] could be addressed. Strong team communication was also seen as a means of mitigating staff confusion about ERAS[21]: specific areas identified as requiring improvement were communications between nurses and surgeons[19], dialogue between staff and patients, in which the compressed and information-filled approach of ERAS can prove especially challenging[24]. Having a small clinical community and a close-knit team was recognised as creating a good basis for effective organisational interactions[22].

Staff also drew attention to the challenges of coordinating the various aspects of the ERAS programme, and maintaining a good collaborative approach to this within the MDT[23]: indeed, there were concerns that a lack of coordination across different clinical departments served to jeopardise ongoing consistency of practice[22], and it was felt that the provision of feedback and audits to hospital stakeholders[20] was a valuable communicative resource in this respect.
For staff working as champions, building good relationships in and across participating ERAS centres was essential for the successful integration of the programme. They recognised that such relationships served to encourage communication about - and, thereby, establish better shared understandings of current practices on the ground[20], and raise awareness about ERAS guidelines[22] by making sure that everyone is onboard. It was felt that ERAS programmes were most effectively introduced using a bottom up, as opposed to a top down approach[20]. Champions indicated that staff were more likely to engage positively with the integration of ERAS practices

For peer review only - http://bmjopen.bmj.com/site/about/guidelines.xhtml

where they are able to be involved in co-creating them from the ground up, since this collaborative endeavour helped to foster a collective sense of responsibility[20].

Theme 2: Resistance to change

Data from the studies included in this review highlighted how *resistance to change* amongst staff had presented a major challenge to the implementation of ERAS at both collective and individual levels. It was noted, for instance, that introducing and implementing the programme requires a culture change[19,20] for staff, which they expect to find big and dramatic[23]. Concerns about the unfamiliarity of new working practices can lead to negative attitudes and a reluctance to engage with ERAS guidelines[23], whilst a fundamental dislike of change more widely also provokes disinclination[19,22]. The scope and intensity of the resistance described here is also motivated by staff age and experience[21]. Newer nurses, for instance, found it easier to adjust to the programme and tended to do so more quickly that those who were seen to be stuck in old ways[23].

Appointing a "champion" was recognised as having been extremely helpful in terms of encouraging positive attitudes and effective collaboration when implementing ERAS programmes[19,20,26]. Staff taking up this, or a similar, role were appointed from a range of MDT disciplines, and included a ward based designated ERAS nurse[23] and an ERAS coordinator[21]. From the perspective of the champions themselves, meanwhile, resistance was conceptualised less broadly and in more precise terms: attributing this, for instance, to a lack of agreement about specific interventions rather than wider processes[20]. They also felt that even where MDTs were, on the whole, easily accepting of ERAS guidelines, there could still be individual level resistance[20] from some staff.

Theme 3: Role and significance of protocol based care

Staff recognised that working to evidence based guidelines and related protocols can in principle be helpful, because doing so ''provides a framework to optimise patient flow by examining what should

For peer review only - http://bmjopen.bmj.com/site/about/guidelines.xhtml

be done, when, and by whom, thereby reducing delays for patients''[23: p.30] standardising practices, reducing variations in treatment, and thereby ostensibly improving the quality of patient care. In practice, however, there were mixed feelings amongst MDT staff as to whether or not this was the case in relation to delivering ERAS interventions. Surgeons felt that these were easily implementable as long as they were based on best evidence and incorporated in standardised order sets[19], whilst anaesthesiologists acknowledged that although they were not currently following a standardised protocol, they were open to the idea of implementing standardised guidelines[19]. There was also agreement amongst MDT staff that the implementation of the ERAS programme would provide consistency across working practices[22].

The studies highlighted several challenges of "fittingness" in relation to ERAS programmes, emphasising the relevance of institutional, organisational and patient factors. Champions noted that ERAS pathways are not definitively prescriptive, and that this leads to variability in how they ultimately become integrated into and operationalised within a site's existing clinical systems[20]. One study found that needing to modify or deviate from ERAS protocols could create confusion for staff[21]. Difficulties in fitting high numbers of patients into the timescales recommended for length of hospital stay under ERAS were also cited as a challenge. Nursing staff seemed to experience the greatest impact of these particular challenges on their day to day work, in which they were faced with the reality that some patients do not and cannot comply with ERAS requirements and do not "fit" standard care trajectories, because they are too frail and old, or have very high levels of comorbidity, and are simply too unwell[21,25]. Such issues presented ethical as well as logistical difficulties for nursing staff. Some described feeling highly conflicted about the tensions they experienced in striving to achieve the standardised care targets of ERAS protocols whilst also upholding their ideals of nursing practice[25]. They felt that they were having to make compromises in their work, and experienced this as a struggle. Particular concerns were raised about the detrimental impact that this was having upon nurses' capacity for providing adequately

For peer review only - http://bmjopen.bmj.com/site/about/guidelines.xhtml

individualised care for patients[25], and the notion of having one protocol for all[23] was felt to be unsatisfactory. Nursing staff felt that the absence of clear guidance about when and how to default or deviate from ERAS protocols led them to be overly cautious in their work, and they indicated that better defined and more precise inclusion criteria about which patients to drive through recovery would be helpful[23].

Theme 4: Knowledge and expectations

Staff recognised that a good knowledge and understanding of ERAS is crucial if it is to be successfully implemented, although the scope of this requirement transcends the procedural details and pragmatic instructions provided by ERAS protocols themselves. Rather, it was important for staff to have a good grasp of its wider aims and objectives[23], and to believe in the value and (potentially) positive impact of the intervention[20,23]. Three of the studies found that, on the whole, staff did feel positive about and favourable towards the implementation of ERAS[19,20,22], and one study showed that although staff were sceptical about it prior to implementation, they felt more positive having seen how well ERAS worked in practice[24]. In all the studies, however, staff acknowledged that considerable challenges still exist and that these will need to be overcome. The nature of such concerns varied for staff, depending upon their own MDT specialty, since this had impact upon the way in which they engaged with ERAS practices in their everyday work. Nurses, in particular, described feeling cautious and sceptical about implementing ERAS because of a lack of confidence, indecision, and anxieties about being challenged by other members of the MDT during ward rounds. They were also worried about any potentially adverse consequences for patients of progressing their recovery in accordance with ERAS[23]. Tacit knowledge was also understood to be important for nurses for their role in implementing ERAS: this helped them to take a common sense[23] approach to the process, especially in terms of knowing when it was appropriate to deviate from ERAS guidelines[23,25].

For peer review only

Setting and effectively managing expectations was a key concern for health professionals in helping them to build shared understandings around ERAS, and to understand their own individual tasks and responsibilities. The expectations of both professionals and patients (and negotiations of the two) were relevant here. Staff felt that they themselves benefitted from setting clear patient expectations[19], and were also keenly aware of some of the complex difficulties in collective understandings of what was expected from whom, when, and in which ways across the MDT, where various parties ‘’made an effort to fulfil the other’s expectations in the situation, but from different perspectives and different understandings of the same situation’’[24: p.420].

Pearsall et al.[19] note that staff expectations - of self and others - differ across the MDT and, importantly, explore how these are linked to (sometimes incorrect) assumptions made by some staff about the knowledge and expectations of their colleagues, creating uncertainty and ambivalence around ERAS implementation. For instance, where nurses anticipated that some surgeons might resist ERAS recommendations, surgeons thought that nursing culture and lack of nursing time would present a problem. Anaesthetists, meanwhile, were concerned that patients would not understand ERAS guidelines and procedures, and assumed that it would be very difficult to amend existing and well-established nursing culture and surgeon behaviours. The surgeons themselves were unconvinced as to whether changes made in accordance with ERAS would make any difference to patients’ experiences of the surgical pathway.

Staff acknowledged that their expectations about ERAS timeframes should be realistic[23], that is, accepting of the reality that some patients would be unable to achieve recovery according to the goals prescribed in the protocol. Whilst some nurses conceptualised such non-achievement as a failure of the [ERAS] programme[23], however, others saw the patients themselves as being responsible for this, on account of them being unprepared for a short hospital stay or early mobilisation, and feeling disproportionately anxious about the process[25]. Staff recognised the

For peer review only - http://bmjopen.bmj.com/site/about/guidelines.xhtml

extent to which good pre-operative education is helpful for patients, but noted that they nevertheless have to deal with problems arising where patients have unrealistic expectations, forget important information, or simply will not comply with ERAS instructions[21]. It was also felt that some patients might be unable to understand the information and instructions that they received, creating difficulties for MDT staff[22].

Theme 5: Temporality

Temporality is highlighted as being important in findings from the studies: the successful embedding of ERAS is described, for instance, as a process that requires a slow but steady approach, becoming normalised over time, until it is established as the standard of care [so that] there's nothing to talk about[20]. The attitudes of staff towards ERAS programmes also changed over time, typically becoming more favourable as the integration continued[24], and nursing staff were keen to reflect on this, recognising how long it had taken them, to see ERAS practices as routine and second nature[23]. Some of the primary challenges identified to the effective ongoing integration of ERAS, meanwhile, were factors relating to a perceived lack of the resources (within and external to local sites) required to maintain the programme over time. These included a lack of adequate finances, limited space, a shortage of equipment, and too few nursing staff[22], as well as poor administrative support for the programme[20]. Insufficient community support for discharge procedures and arrangements was also cited as a hindrance to successful embedding of ERAS. Temporality also relates in part to the point at which data was collected in the included studies. This means that the different findings they report also reflect the different stages of ERAS implementation being studied, i.e. before, during or after implementation of the ERAS programme [19].

DISCUSSION

For peer review only - http://bmjopen.bmj.com/site/about/guidelines.xhtml

Our meta-synthesis of qualitative studies produced five themes, which reflect key considerations described by health professionals in relation to their experiences of delivering ERAS pathways. These themes were communication and collaboration, resistance to change, role and significance of protocol-based care, knowledge and expectations, and temporality. Staff emphasised that there must be effective MDT collaboration and communication, if ERAS practices are to be successfully implemented and integrated. This included providing a thorough education to staff and patients about ERAS, and ensuring that information and knowledge about it was clearly and consistently disseminated across the MDT. The coordination of ERAS approaches was acknowledged to be challenging, and the appointment of a designated ERAS champion was experienced as being helpful in this respect.

The value of evidence based guidelines was described as useful means of helping to improve patient care by bringing about a standardisation of practices and a reduction in variations in treatment, but staff were ambivalent about the extent to which ERAS created such consistencies in practice. Concerns were raised about the necessity of modifying or deviating from ERAS guidelines, where these did not “fit” with local site systems or with the care requirements of individual patients. A need for more precise information about how best to do this was identified.

A comprehensive knowledge and understanding of ERAS was cited as being essential to its successful implementation: in terms of both procedural detail and the broader aims and objectives that underpin the intervention itself. Staff were concerned about the impact of ERAS upon their own everyday working practices, and in relation to their own speciality within the MDT. Staff expectations about ERAS varied across MDT disciplines, and the need to set and manage these effectively was prioritised. The importance of establishing ‘realistic’ expectations was emphasised for staff and also the patients for whom they care. This is a key finding that underpins the need for clear guidance to staff who are delivering ERAS.

For peer review only - http://bmjopen.bmj.com/site/about/guidelines.xhtml

The implementation and embedding of ERAS was understood to require complex processes of adjustment, acceptance and engagement for staff, constituting a process that evolves gradually over time. Staff attitudes towards ERAS were also subject to temporal change, and tended to become increasingly favourable via reflections upon how well the new and or amended practices were working, and the ways in which they became 'normalised'.

Given that ERAS seeks to improve patients' outcomes through consistency in care, findings from our review highlight that, whilst health professionals are confident that ERAS pathways have the potential to achieve this, some key improvements are needed. The findings of this review are new because they highlight key and common themes that appear in all delivery of ERAS in diverse contexts. They also build upon existing knowledge about ERAS by showing that the pathway is implemented disparately across different settings, according to local contexts and circumstances[4], and that the provision of better information and education to staff and patients can achieve better consistency. Our review also indicates that health professionals cite resistance to change amongst staff as a hindrance to the effective implementation of ERAS[6], Our findings demonstrate that effective collaboration and communication amongst staff – and between staff and patients – helps to improve the effectiveness of ERAS[5] and, again, good clear guidance could help with this. This review also observes that staff use different strategies of discursive framing to describe their experiences of implementing and engaging with ERAS practices, which indicate that temporality is an important factor in this respect. These include accounts of adapting to, adjusting to, and coming around to the programme[23], adopting the pathway[19], being accepting of it[20], and compliance with ERAS guidelines[21]. From a discursive psychological perspective – which focuses upon social construction through language[27], these framings illustrate how the implementation of ERAS is produced through talk[28] as a gradual process which evolves over time. The most important finding from the included studies is that appointing a dedicated Enhanced Recovery "champion" helps to

For peer review only - http://bmjopen.bmj.com/site/about/guidelines.xhtml

mitigate many of the barriers to the effective implementation of ERAS [7]. The studies indicate that this improves MDT communication and collaboration, assists the provision of consistent information and education to staff and patients, and helps to alleviate resistance to change and lack of confidence amongst staff when they are faced with new working practices brought about by ERAS protocols. The is the key implication of this review, and an important message for future practice.

We conducted the systematic review in a manner that was designed to capture as many studies as possible by using keywords that were identified and refined from existing literature. To enhance rigour in study selection the included studies were all appraised by the two authors. This process acted as a screening process that allowed us to exclude three studies and retain eight as well as appraise whether the included studies sufficiently addressed the ten questions from the CASP qualitative checklist. Assessment using the CASP checklist can be conducted in a variety of ways and our process enabled us to define all studies to be of sufficient quality. To improve reporting quality of this review, we have adhered to the ENTREQ guidance on the reporting of qualitative syntheses[29]. The reflexive approach of the authors in the selection process sought to minimise researcher bias.

One of the strengths of this review is that it includes a range of different studies, and therefore incorporates a variety of populations and geographical contexts. Further strengths are the diversity of methodological approaches used in the studies, and the different clinical contexts and local environments of the included studies. This provides a richness of perspectives. Additionally, there are few existing systematic reviews of qualitative studies on staff experiences of ERAS. This paper therefore makes a valuable contribution to the field of literature. A limitation of this review is the small number of included studies, however, we included studies in six countries across four surgical specialities and as such our work highlights key issues that are transferable between contexts. Another limitation is that there are no ethnographic studies included in our review and, as such, it

For peer review only - http://bmjopen.bmj.com/site/about/guidelines.xhtml

does not benefit from the use of observational data on the implementation of ERAS. We suggest that future research could build upon existing knowledge of and understanding about staff perspectives of ERAS by taking an ethnographic approach. We also note that ERAS pathways are now being implemented in elective orthopaedic surgery, and suggest that this is a valuable area for future study.

CONCLUSION

We reviewed and synthesised qualitative studies that explore health professionals' experiences of and perspectives on the enhanced recovery after surgery (ERAS) pathway. This is the first systematic review to draw together findings from qualitative studies with health professionals, and to inform implementation of ERAS we would argue that their experiences and views are crucial. Findings from our review indicate that, whilst staff generally feel positive about the implementation of ERAS, they acknowledge that the process is complex and challenging. Many of the challenges identified, such as resistance to change and lack of confidence can however be mitigated by ensuring that MDTs understand ERAS principles and guidelines, and that they communicate well with one another and with patients. Other challenges, such as a lack of local resources and high rates of comorbidity amongst patients are perhaps more challenging to address. We suggest that the provision of comprehensive, coherent and locally relevant information to health professionals would help to improve the implementation and delivery of ERAS pathways. Identifying and recruiting an ERAS champion is also recommended as means of improving the effectiveness of the pathway.

A: CONTRIBUTORSHIP STATEMENT

The authors of this article are Dr Rachel Cohen (RC) and Professor Rachael Gooberman-Hill (RGH). Both authors made substantial contributions to the conception and the design of the systematic review. Literature searches were conducted by RC, and RGH carried out the CASP screening. Both RC and RGH contributed to the extraction, analysis and interpretation of data from the papers included

For peer review only - http://bmjopen.bmj.com/site/about/guidelines.xhtml

in the review. RC and RGH worked on drafts of the review, made revisions and agreed on a final version for publication. Both RC and RGH agree to be accountable for all aspects of the work in ensuring that questions related to the accuracy or integrity of any part of the work are appropriately investigated and resolved.

Support for the study was received from the Oxford NIHR Biomedical Research Centre, Nuffield Orthopaedic Centre, University of Oxford. The views and opinions expressed therein are those of the authors and do not necessarily reflect those of the HS&DR Programme, NIHR, NHS or the Department of Health. We thank the wider team for the study, including Professor Andrew Judge as Principal Investigator. We also thank Christine Hobson for her work in support of our research.

B: COMPETING INTERESTS

The author and co-author have no competing interests to declare

C: FUNDING

This review forms part of the dissemination strategy for the Atlas (Ethnographic study of care pathways for hip and knee replacement) project, which is funded by the National Institute for Health Research Health Services and Delivery Research Programme (project number 14/46/02).

D: DATA SHARING STATEMENT

Data for the study may be made available from University of Bristol's research data repository under a controlled access arrangement. Requests for access will be referred to the University’s data access committee before data can be shared under a data sharing agreement. As such, anonymous data from the study may be seen and used by other researchers, for ethically approved research projects, on the understanding that

For peer review only

For peer review only - http://bmjopen.bmj.com/site/about/guidelines.xhtml

confidentiality will be maintained. Release of the data will be at the discretion of the data access committee (data custodian).

REFERENCES

1. Kehlet H. Fast-track colorectal surgery. *The Lancet* 2008;371:791-3.

2. Department of Health. Enhanced Recovery Partnership Programme Project Report. March 2011.Available from: https://www.gov.uk/government/publications/enhanced-recovery-partnership-programme (accessed 1 Feb 2017).

3. Campbell NC, Murray E, Darbyshire J, *et al*. Designing and evaluating complex interventions to improve health care. *BMJ* 2007;334:455-9.

For peer review only - http://bmjopen.bmj.com/site/about/guidelines.xhtml

4. Craig P, Dieppe P, Macintyre S, *et al.* Developing and Evaluating Complex Interventions: The New Medical Research Council Guidance. *BMJ* 2008;337:979-83.

5. Waring J, Marshall F, Bishop S, *et al*. An ethnographic study of knowledge sharing across the boundaries between care processes, services and organisations: the contributions to 'safe' hospital discharge. *Health Serv Deliv Res* 2014:2;1-160.

6. Moore G, Audrey S, Barker M, *et al*. Process evaluation in complex public health intervention studies: the need for guidance. *J Epidemiol Community Health* 2014;68:101-2.

7. Paton F, Chambers D, Wilson P, *et al.* Effectiveness and implementation of enhanced recovery after surgery programmes: a rapid evidence synthesis. *BMJ Open* 2014;4:e005015. doi:10.1136/bmjopen-2014-005015.

8. Bernard H, Foss M. The impact of the enhanced recovery after surgery (ERAS) programme on community nursing. *Br J Community Nurs* 2014;19:184-8.

9. Blazeby JM, Soulsby M, Winstone K, *et al*. A qualitative evaluation of patients' experiences of an enhanced recovery programme for colorectal cancer. *Colorectal Dis* 2010;12:e236-42.

10. Hunt GR, Hall GM, Murthy BV, *et al*. Early Discharge Following hip Arthroplasty: Patients' acceptance masks doubts and concerns. *Health Expect* 2009;12:130-7.

11. Husted H, Holm G. Fast track in total hip and knee arthroplasty - experiences from Hvidovre University Hospital, Denmark. *Injury* 2006;37(Suppl 5):S31-S35.

For peer review only - http://bmjopen.bmj.com/site/about/guidelines.xhtml

12. Norlyk A. The experience of fast track postoperative rehabilitation regimen: the patients' perspective. *Klinisk Sygepleje* 2008;22:53-63.

13. Norlyk A, Harder I. After colonic surgery: The lived experience of participating in a fast-track programme. *Int J Qual Stud on Health Well-being* 2009;4:170-180.

14. Sibbern T, Bull Sellevold V, Steindal SA, *et al*. Patients' Experiences of Enhanced Recovery after Surgery: a systematic review of qualitative studies. *J Clin Nurs* 2017;26:1172-188.

15. Critical Skills Appraisal Programme. *CASP Qualitative Checklist*. Available from: http://www.casp-uk.net/checklists (accessed 30 May 2018).

16. Polit DF, Beck CT. Nursing Research: Generating and Assessing Evidence for Nursing Practice. Philadelphia: Wolters Kluwer/Lippincott Williams and Wilkins; 2008.

17. Graneheim UH, Lundman B. Qualitative content analysis in nursing research: concepts, procedures and measures to achieve trustworthiness. *Nurse Educ Today* 2004;24:105–12.

18. Uhrenfeldt L, Aagaard H, Hall EO, *et al.* A qualitative meta-synthesis of patients' experiences of intra- and inter-hospital transitions. *J Adv Nurs* 2013;69:1678–90.

19. Pearsall EA, Meghiji A, Pitzul KB, *et al*. A Qualitative Study to Understand the Barriers and Enablers in Implementing an Enhanced Recovery after Surgery Program. *Ann Surg* 2015;261:92-6.

For peer review only

For peer review only - http://bmjopen.bmj.com/site/about/guidelines.xhtml

20. Gotlib Conn L, McKenzie M, Pearsall EA, *et al*. Successful implementation of an enhanced recovery after surgery programme for elective colorectal surgery: a process evaluation of champions' experiences. *Implement Sci* 2015;10:99.

21. Lyon A. Solomon MJ, Harrison JD. A Qualitative Study Assessing the Barriers to Implementation of Enhanced Recovery after Surgery. *World J Surg* 2014;38:1374-80.

22. Alawadi ZM, Leal I, Phatak UR, *et al*. Facilitators and barriers of implementing enhanced recovery in colorectal surgery at a safety net hospital: A provider and patient perspective. *Surgery* 2016;159:700-12.

23. Jeff A, Taylor C. Ward nurses' experiences of enhanced recovery after surgery: a grounded theory approach. *Gastrointestinal Nursing* 2014;12:23-31.

24. Wagner L, Carlslund AM, Moller C, *et al.* Patient and staff (doctors and nurses) experiences of abdominal hysterectomy in accelerated recovery programme: A qualitative study. *Dan Med Bull* 2004;51:418-21.

25. Berthelsen CB, Frederiksen K. Orchestrating care through the fast track perspective: A qualitative content analysis of the provision of individualised nursing care in orthopaedic fast-track programmes. *Int J Orthop Trauma Nurs* 2017;24:40-9.

26. Sjetne IS, Krogstad U, Odegard, S, *et al*. Improving quality by introducing enhanced recovery after surgery in a gynaecological department: consequences for ward nursing practice. Qual Saf Health Care; 2009:18;236-40.

For peer review only - http://bmjopen.bmj.com/site/about/guidelines.xhtml

For peer review only

27. Wiggins S, Potter J. Discursive Psychology. In: Willig C, Hollway SR, eds. *Handbook of Qualitative Research*. London:Sage 2008:72-89.

28. Potter J, Wetherell M. *Discourse and Social Psychology: Beyond Attitudes and Behaviour.* London: Sage, 1987.

29. Tong A, Flemming K, McInnes *et al*. Enhancing transparency in reporting the synthesis of qualitative research: ENTREQ. *BMC Med Res Methodol* 2012;12:181.

For peer review only

For peer review only - http://bmjopen.bmj.com/site/about/guidelines.xhtml

PRISMA 2009 Flow Diagram
Staff experiences of Enhanced Recovery after Surgery – Systematic Review of Qualitative Studies

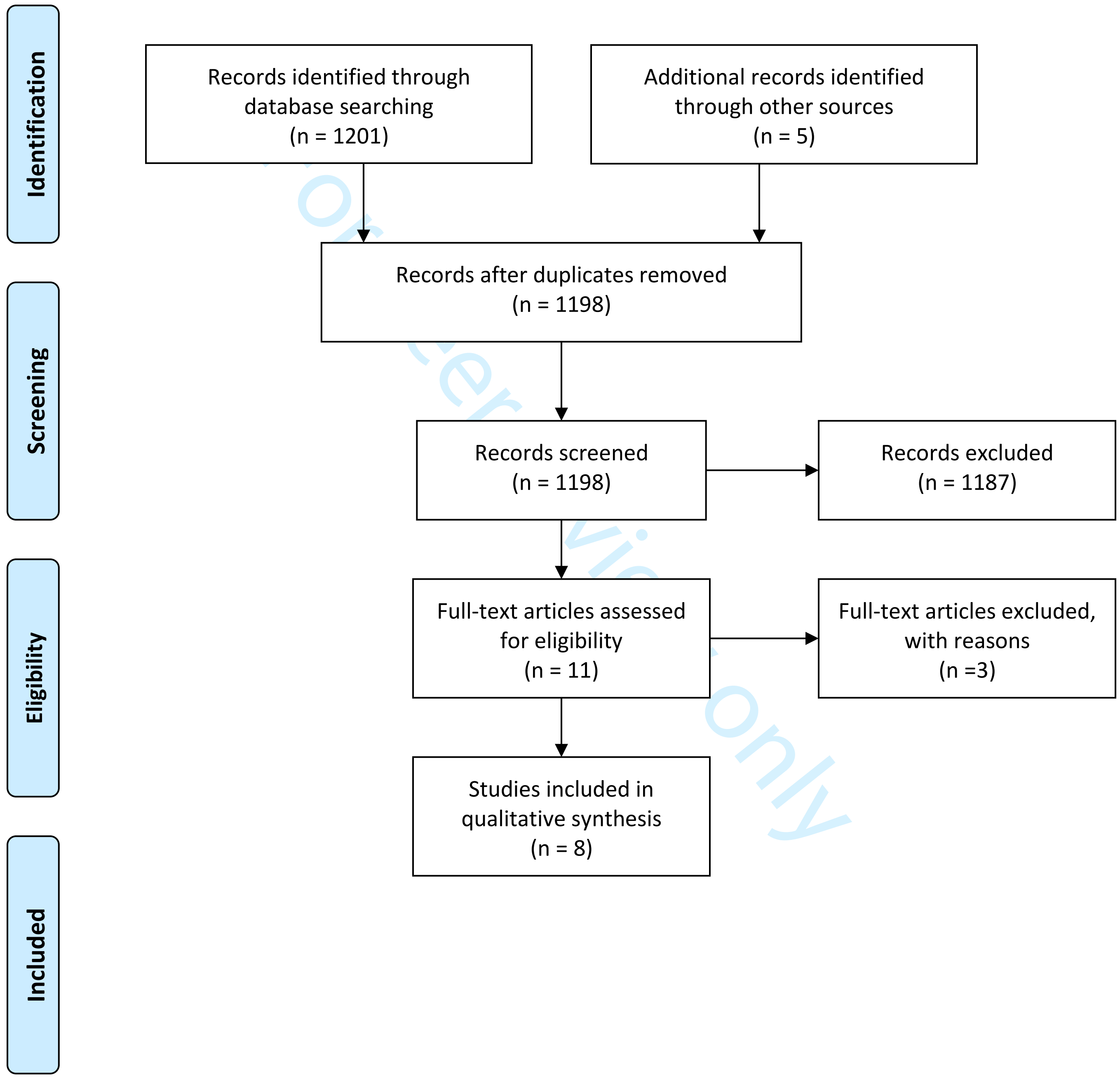

From: Moher D, Liberati A, Tetzlaff J, Altman DG, The PRISMA Group (2009). *P*referred *R*eporting *I*tems for *S*ystematic Reviews and *M*eta-*A*nalyses: The PRISMA Statement. PLoS Med 6(7): e1000097. doi:10.1371/journal.pmed1000097

For more information, visit www.prisma-statement.org.

PRISMA 2009 Checklist

Section/topic	#	Checklist item	Reported on page #
**TITLE**			
Title	1	Identify the report as a systematic review, meta-analysis, or both.	1
**ABSTRACT**			
Structured summary	2	Provide a structured summary including, as applicable: background; objectives; data sources; study eligibility criteria, participants, and interventions; study appraisal and synthesis methods; results; limitations; conclusions and implications of key findings; systematic review registration number.	2
**INTRODUCTION**			
Rationale	3	Describe the rationale for the review in the context of what is already known.	4
Objectives	4	Provide an explicit statement of questions being addressed with reference to participants, interventions, comparisons, outcomes, and study design (PICOS).	5
**METHODS**			
Protocol and registration	5	Indicate if a review protocol exists, if and where it can be accessed (e.g., Web address), and, if available, provide registration information including registration number.	5
Eligibility criteria	6	Specify study characteristics (e.g., PICOS, length of follow-up) and report characteristics (e.g., years considered, language, publication status) used as criteria for eligibility, giving rationale.	6
Information sources	7	Describe all information sources (e.g., databases with dates of coverage, contact with study authors to identify additional studies) in the search and date last searched.	5,6
Search	8	Present full electronic search strategy for at least one database, including any limits used, such that it could be repeated.	5,6
Study selection	9	State the process for selecting studies (i.e., screening, eligibility, included in systematic review, and, if applicable, included in the meta-analysis).	5,6,7
Data collection process	10	Describe method of data extraction from reports (e.g., piloted forms, independently, in duplicate) and any processes for obtaining and confirming data from investigators.	6-9
Data items	11	List and define all variables for which data were sought (e.g., PICOS, funding sources) and any assumptions and simplifications made.	6-9
Risk of bias in individual studies	12	Describe methods used for assessing risk of bias of individual studies (including specification of whether this was done at the study or outcome level), and how this information is to be used in any data synthesis.	6-9
Summary measures	13	State the principal summary measures (e.g., risk ratio, difference in means).	N/A
Synthesis of results	14	Describe the methods of handling data and combining results of studies, if done, including measures of consistency (e.g., I^2) for each meta-analysis.	6-9

For peer review only - http://bmjopen.bmj.com/site/about/guidelines.xhtml

PRISMA 2009 Checklist

Section/topic	#	Checklist item	Reported on page #
Risk of bias across studies	15	Specify any assessment of risk of bias that may affect the cumulative evidence (e.g., publication bias, selective reporting within studies).	N/A
Additional analyses	16	Describe methods of additional analyses (e.g., sensitivity or subgroup analyses, meta-regression), if done, indicating which were pre-specified.	N/A
**RESULTS**			
Study selection	17	Give numbers of studies screened, assessed for eligibility, and included in the review, with reasons for exclusions at each stage, ideally with a flow diagram.	6,7
Study characteristics	18	For each study, present characteristics for which data were extracted (e.g., study size, PICOS, follow-up period) and provide the citations.	10-12
Risk of bias within studies	19	Present data on risk of bias of each study and, if available, any outcome level assessment (see item 12).	N/A
Results of individual studies	20	For all outcomes considered (benefits or harms), present, for each study: (a) simple summary data for each intervention group (b) effect estimates and confidence intervals, ideally with a forest plot.	N/A
Synthesis of results	21	Present results of each meta-analysis done, including confidence intervals and measures of consistency.	N/A
Risk of bias across studies	22	Present results of any assessment of risk of bias across studies (see Item 15).	N/A
Additional analysis	23	Give results of additional analyses, if done (e.g., sensitivity or subgroup analyses, meta-regression [see Item 16]).	N/A
**DISCUSSION**			
Summary of evidence	24	Summarize the main findings including the strength of evidence for each main outcome; consider their relevance to key groups (e.g., healthcare providers, users, and policy makers).	19-21
Limitations	25	Discuss limitations at study and outcome level (e.g., risk of bias), and at review-level (e.g., incomplete retrieval of identified research, reporting bias).	21,22
Conclusions	26	Provide a general interpretation of the results in the context of other evidence, and implications for future research.	21,22
**FUNDING**			
Funding	27	Describe sources of funding for the systematic review and other support (e.g., supply of data); role of funders for the systematic review.	23

From: Moher D, Liberati A, Tetzlaff J, Altman DG, The PRISMA Group (2009). Preferred Reporting Items for Systematic Reviews and Meta-Analyses: The PRISMA Statement. PLoS Med 6(7): e1000097. doi:10.1371/journal.pmed1000097

For more information, visit: **www.prisma-statement.org**.

For peer review only - http://bmjopen.bmj.com/site/about/guidelines.xhtml

BMJ Open

Staff Experiences of Enhanced Recovery after Surgery – Systematic Review of Qualitative Studies

Journal:	*BMJ Open*
Manuscript ID	bmjopen-2018-022259.R2
Article Type:	Research
Date Submitted by the Author:	09-Oct-2018
Complete List of Authors:	Cohen, Rachel; University of Bristol, School of Population Health Sciences<br>Gooberman-Hill, Rachael; University of Bristol, School of Clinical Sciences
**Primary Subject Heading**:	Health services research
Secondary Subject Heading:	Qualitative research
Keywords:	Enhanced recovery, Qualitative, Joint replacement

SCHOLARONE™ Manuscripts

Article Title: STAFF EXPERIENCES OF ENHANCED RECOVERY AFTER SURGERY – SYSTEMATIC REVIEW OF QUALITATIVE STUDIES

Authors: #Dr Rachel Cohen and Professor Rachael Gooberman-Hill*

***Corresponding author:** Dr Rachel Cohen, Centre for Academic Mental Health, Department of Population Health Sciences, Bristol Medical School, University of Bristol, Bristol, BS8 2BN, UK

Corresponding author's Email address: rachel.cohen@bristol.ac.uk Tel: +44(0)117 331 4007

#Co-author: Professor Rachael Gooberman-Hill, Professor of Health and Anthropology, Translational Health Sciences, Bristol Medical School, University of Bristol, Learning and Research Building, Level 1, Southmead Hospital, Bristol BS10 5NB, UK

For peer review only

Keywords: Enhanced Recovery, qualitative, joint replacement

For peer review only - http://bmjopen.bmj.com/site/about/guidelines.xhtml

ABSTRACT

Title: Staff Experiences of Enhanced Recovery after Surgery – Systematic Review of Qualitative Studies

Objectives: To conduct a systematic review of qualitative studies which explore health professionals' experiences of and perspectives on the enhanced recovery after surgery (ERAS) pathway.

Design: Systematic review of qualitative literature using a qualitative content analysis. Literature includes the experiences and views of a wide range of Multidisciplinary Team and Allied Health Professional staff, to incorporate a diverse range of clinical and professional perspectives.

Data sources: PsychINFO, Medline, Cinahl, and PubMed were searched in May 2017

Eligibility criteria for selecting studies: The searches included relevant qualitative studies across a range of healthcare contexts. We included studies published from 2000-2017, as an appropriate timeframe to capture evidence about ERAS after implementation in the late 1990s. Only studies published in the English language were included, and we included studies that explicitly stated that they used qualitative approaches

Data extraction and synthesis: Literature searches were conducted by the first author and checked by the second author: both contributed to the extraction and analysis of data. Studies identified as relevant were assessed for eligibility using the Critical Appraisal Skills Programme (CASP) guidance.

Results: Eight studies were included in the review, including studies in six countries and in four surgical specialties. Included studies focus on health professionals' experiences of ERAS before, during and after implementation in colorectal surgery, gastrointestinal surgery, abdominal hysterectomy, and orthopaedics. Five main themes emerged in the analysis: communication and collaboration, resistance to change, role and significance of protocol-based care, and knowledge and expectations. Professionals described the importance of effective multidisciplinary team collaboration and communication, providing thorough education to staff and patients, and appointing a dedicated champion as means to implement and integrate ERAS pathways successfully. Evidence based guidelines were thought to be useful for improvements to patient care by

For peer review only - http://bmjopen.bmj.com/site/about/guidelines.xhtml

standardising practices and reducing treatment variations, but were thought to be too open to interpretation at local levels. Setting and managing ‘realistic’ expectations of staff was seen as a priority. Staff attitudes towards ERAS tend to become more favourable over time, as practices become successfully ‘normalised’. Strengths of the review are that it includes a wide range of different studies, a variety of clinical populations, diversity of methodological approaches and local contexts. Its limitation is the inclusion of a small number of studies, although these represent six countries and four surgical specialties, and so our findings are likely to be transferable.

Conclusions: Staff feel positive about the implementation of ERAS, but find the process is complex and challenging. Challenges can be addressed by ensuring that multidisciplinary teams understand ERAS principles and guidelines, and communicate well with one another and with patients. Provision of comprehensive, coherent and locally relevant information to health professionals is helpful. Identifying and recruiting local ERAS champions is likely to improve the implementation and delivery of ERAS pathways.

ARTICLE SUMMARY

Strengths and limitations of this study

- There is a need to synthesise qualitative evidence about staff experiences of Enhanced Recovery After Surgery (ERAS) because these provide insight into implementation of ERAS
- The review includes studies with a diverse range of populations, contexts and methodological approaches
- The review includes a small number of studies, but includes studies from six countries in four surgical specialties and so the findings are likely to be transferable and of relevance to several contexts of ERAS implementation

For peer review only - http://bmjopen.bmj.com/site/about/guidelines.xhtml

INTRODUCTION

Enhanced recovery after surgery (ERAS) programmes were introduced and began to be implemented in the late 1990s[1], as part of an initiative towards reducing variations in patient care and improving quality standards[2]. Building upon their Danish origins, ERAS programmes have been internationally adopted, and widely implemented for major elective surgical pathways in colorectal surgery, orthopaedics, gynaecology, cardiology and urology. Depending upon the kind of diagnostic and surgical care in question, ERAS programmes are sometimes referred to using different names, including 'fast-track surgery', 'rapid recovery', 'accelerated discharge' or 'early discharge'.
The aim of ERAS pathways is to reduce length of hospital stay and lessen readmissions, minimise surgical complications, decrease morbidity, and improve cost effectiveness. Best described as a complex intervention[3,4], ERAS seeks to improve patient experiences and outcomes by focusing on key aspects of the care pathway, pre-, peri- and post operatively, as a means of reducing physiological and psychological stress. This involves the provision of better education and information for patients prior to their operations, the use of minimally invasive surgical techniques and anaesthesia, optimal pain management and early post-operative mobilisation, as well as the preparation of a discharge plan[5].

Despite their protocol-based foundations, evidence from recent studies indicates that ERAS pathways are implemented variably across different hospital settings. More information is needed about what the core active ingredients of ERAS are. We also need to know more about how these ingredients exert their effect according to local circumstances, and about how they shape (and are shaped by) the context of their implementation[4,6]. Existing literature has drawn particular attention to the factors which help, and those which hinder, the successful implementation of ERAS, identifying important barriers and facilitators to the process Barriers include resistance to change, inadequate funding, lack of support from management, high staff turnover, poor documentation,

For peer review only - http://bmjopen.bmj.com/site/about/guidelines.xhtml

and shortness of time, whilst facilitators included a dedicated enhanced recovery lead, effective multidisciplinary team working and ongoing education for staff and patients[7].

Patient experiences of and satisfaction with ERAS pathways have been studied using both quantitative and qualitative approaches: the latter have been especially useful in improving understandings of patient experiences and perspectives e.g.[8,9,10,11,12,13]. Sibbern et al's[14] systematic review of studies of patients' experiences provides a comprehensive discussion of existing qualitative research on this specific topic. Health professionals' satisfaction with and perspectives on ERAS, meanwhile, have typically been explored using quantitative approaches. Information about the experiences of health professionals in delivery of ERAS is needed to inform implementation and healthcare policy and practice. Such experiences are best gathered in details through qualitative research.

This article describes a systematic review of qualitative studies of health professionals' experiences of ERAS pathways. The aim of the review was to synthesise evidence of the experience of health professionals who have been involved in implementing the ERAS programme, incorporating their experiences before, during and after the programme was implemented, and of its subsequent delivery. The review aims to identify overarching themes that provide opportunities for improving implementation and practice.

METHODS

Patient and Public Involvement

This paper is a systematic review of qualitative studies. No patients were involved in the review.

For peer review only - http://bmjopen.bmj.com/site/about/guidelines.xhtml

For peer review only

Prospero registration

Our systematic review was registered on PROSPERO in 2017: the registration number is CRD42017059952. The review sought to describe the experiences and perspectives of healthcare professionals involved in delivering enhanced recovery pathways.

Literature search

We used methods of systematic search and review and conducted a search of PsychINFO, Medline, Cinahl, and PubMed to identify relevant qualitative studies across a range of health care contexts. The searches were conducted by the first author and checked by the second author. The searches included studies published from 2000-2017, as an appropriate timeframe to capture evidence about ERAS after implementation in the late 1990s. Only studies published in the English language were included, and we included studies that explicitly stated that they used qualitative approaches. For all of the databases, the search terms used were:

> ERAS OR enhanced recovery OR fast-track OR accelerated recovery OR rapid recovery OR early discharge OR patients discharge OR enhance* recov* after surg*
>
> Staff perspective OR staff experience* OR staff perception* OR ward staff OR nurs* OR professional*
>
> Qualitative OR interview* OR ethnograph* OR observation

The reference lists of articles identified from the database search were also scrutinised for possible additional studies.

Quality assessment

As shown in the PRISMA flowchart (supplementary file), the database searches yielded 1201 articles in total. In addition, through searching the reference lists of the included studies, we identified five

For peer review only - http://bmjopen.bmj.com/site/about/guidelines.xhtml

further records. Eleven studies met the inclusion criteria, and were assessed for eligibility using the Critical Appraisal Skills Programme (CASP) guidance (Critical Skills Appraisal Programme 2017)[15]. The CASP checklist for qualitative research provides a means of identifying the strengths and weaknesses of research articles, assessing their usefulness and validity, and their relevance for inclusion in the review. The CASP qualitative checklist was designed as a pedagogic tool and therefore as a means of assessing whether qualitative approaches are appropriate to a research question, the value of results, and to provide the opportunity to assess quality in a qualitative, expertise-based and discursive fashion. Therefore, we considered the eleven studies using the ten CASP questions which are: aim, methodology, design, recruitment strategy, data collection, relationship between researcher and participants, ethical issues, data analysis, findings and research value - three studies were excluded, and the remaining eight were included. Two of the three that were excluded at this stage were quantitative rather than qualitative, and one focused on rehabilitation following hip and knee arthroplasty, but not specifically on ERAS. The two authors independently conducted quality assessment and agreed that all eight articles addressed all ten CASP criteria and were of sufficient rigour and relevance for inclusion in the review.

Data extraction

After completion of quality assessment we conducted a qualitative meta-synthesis of the eight eligible articles. This comprised close reading and extraction of key findings using descriptive qualitative design[16], and a qualitative content analysis[17,18]. For the analysis, we focused on the manifest content of the articles, i.e. what the texts say[17]. This involved searching for the common concepts and themes[18] addressed in the articles regarding health professionals' experiences of and perspectives on ERAS. Supporting quotes were also gathered. This enabled us to develop meaning units within the themes, with the meaning units extracted from the findings of the studies. Meaning units refer to the main considerations in relation to each theme that were raised by staff about their experiences of implementing and delivering ERAS programmes. These were then

For peer review only - http://bmjopen.bmj.com/site/about/guidelines.xhtml

For peer review only

condensed into content related categories, which the authors discussed and agreed upon. Content related categories refer to the suggested techniques for addressing and responding to these considerations. We then synthesised the chosen categories into themes as shown in Table 1.

For peer review only

For peer review only - http://bmjopen.bmj.com/site/about/guidelines.xhtml

TABLE 1

Theme	Meaning Unit	Content related category
Collaboration and communication	• Staff find the information-rich nature of ERAS confusing. Many staff feel that they do not understand it well enough and/or that they have not received sufficiently clear or consistent information or training<br>• Information about ERAS is not always disseminated between staff – and between staff and patients – in a coherent and consistent way.<br>• Collaborative MDT work is hindered by high staff turnover and a lack of coordination across different departments	• Providing staff and their patients with a comprehensive education about and introduction to ERAS improves understanding and helps to mitigate confusion<br>• Strong team communications help to ensure the effective dissemination of information<br>• Building good relationships within the MDT helps to encourage dialogue between staff, and to improve their willingness and ability to collaborate. The appointment of a dedicated ERAS 'champion' improves staff engagement and collaborative working
Resistance to change	• Staff are reluctant to implement or engage with new and unfamiliar working practices. Some staff – especially those who are older, or more well established in their role – tend to dislike change more generally and are disinclined to engage with ERAS.	• Appointing and ERAS champion helps to encourage more positive attitudes amongst staff
Role and significance of protocol based care	• Staff recognise the usefulness of evidence-based protocol guidelines as a means of reducing variations and standardising practice, but have mixed feelings about whether ERAS facilitates this well.<br>• ERAS is not definitively prescriptive, and therefore allows for too much variability in local implementation.	• The incorporation of standardised order sets and basing ERAS practices on best evidence increases staff willingness to implement it as a complex intervention<br>• Having a local ERAS champion helps to improve consistency in implementing and operationalising the pathway into existing systems at local sites

For peer review only

	• Some staff feel conflicted about having to compromise their capacity for and confidence in providing individualised care for patients.	• Clearer guidance about when it is acceptable to deviate from ERAS protocols would improve staff confidence.
Knowledge and expectations	• Staff feel that they need a broader knowledge and understanding of ERAS, i.e. beyond protocol guidelines.<br><br>• Staff are sceptical about the usefulness and value of ERAS prior to its implementation.<br><br>• Managing the expectations of staff and patients is recognised as being crucial to the successful implementation of ERAS. Differing professional perspectives, which are sometimes based on incorrect assumptions, can create ambivalence and uncertainty amongst staff. Staff use tacit knowledge and a "common sense" approach to overcome this.	• Belief in the value and potential positive impact of ERAS improves the willingness of staff to engage with the pathway and its guidelines.<br><br>• Staff feel more positive about and favourable towards ERAS when they have seen it work successfully in practice.<br><br>• Setting clear and realistic expectations about ERAS helps to improve staff and patient experiences of the pathway.

For peer review only

For peer review only

For peer review only - http://bmjopen.bmj.com/site/about/guidelines.xhtml

RESULTS

The eight studies included were conducted in the UK (n = 1), US (n = 1), Canada (n = 2), Denmark (n = 2), Norway (n=1) and Australia (n = 1) (Table 2).

TABLE 2

Study	Study design	Surgical population	Methodology and methods	Number and type of participants	Country	Key findings
Alawadi et al	Qualitative study to assess the perceived barriers and facilitators before ERAS adoption.	Colorectal surgery	Qualitative interviews with MDT staff and patients. Content analysis	8 anaesthesiologists, 5 surgeons, 6 nurses, and 18 patients	US	Conclusion: 'Although limited hospital resources are perceived as a barrier to ERAS implementation… there is strong support for such pathways and multiple factors were identified that may facilitate change' (2016: 700)
Sjetne et al	Pre-postintervention prospective design, to monitor changes in	Gynaecological surgery	Questionnaires and qualitative interviews.	34, 33 and 32 nurses returned questionnaires in	Norway	Conclusion: 'expected clinical gains achieved by

For peer review only - http://bmjopen.bmj.com/site/about/guidelines.xhtml

	workload and work environment of ward nursing staff when ERAS was introduced		Quantitative data analysed using SAS (t tests and differences in means), qualitative data used to elaborate the topics studied	phases 1,2, and 3 respectively (100% survey response rate)<br>9 interviews with 4 different nurses		introducing ERAS are achieved without compromising the work environment of ward nurses' (2009: 239)
Pearsall et al	Qualitative study to understand barriers and enablers in perioperative implementation of ERAS	Colorectal surgery	Qualitative semi structured interviews. Thematic analysis	19 general surgeons, 18 anaesthesiologists, 18 nurses	Canada	Conclusion: 'participants supported the need for implementation of an ERAS program… [but] felt there remained major barriers to [its] successful implementation' (2015: 96)
Wagner et al	Exploratory and descriptive qualitative study to gather knowledge about staff and patient experiences of the Accelerated Recovery Programme (ARP)	Abdominal hysterectomy	Qualitative individual interviews and focus groups with staff, observation of and interviews with patients. Thematic analysis.	Observation of 17 patients, 10 of whom were interviewed twice Interviews with 15 staff, who all participated in focus groups	Denmark	Conclusion: patients underwent ARP without significant problems, but identified a need for greater psychological support. Staff data showed a positive change in opinion and an

For peer review only - http://bmjopen.bmj.com/site/about/guidelines.xhtml

						understanding of ARP. Recommendations made for better information to be provided to staff and patients, in consultation rooms and outpatient clinics
Jeff et al	To explore and describe ward nurses' experience of ERAS in the postoperative phase	Gastrointestinal surgery	Semi structured interviews and documentary evidence (memos and reflective journals). Thematic analysis.	Interviews with 8 (of a possible 30) nurses	UK	Conclusion: 'the central difficulty experienced by nurses was trying to adapt the protocol to the demands of patient care delivery within the constraints of their role and organisational culture' (2014: 31)
Gotlib Conn et al	Process evaluation of ERAS champions' experiences. To understand enablers and barriers to the successful implementation of ERAS	Colorectal surgery	Qualitative semi structured interviews. Normalisation Process Theory framework analysis.	5 surgeons, 14 anaesthesiologists, 15 nurses, and 14 project coordinators	Canada	Conclusion: successful implementation of ERAS is achieved by a 'complex series of cognitive and social processes… [the study

For peer review only - http://bmjopen.bmj.com/site/about/guidelines.xhtml

						demonstrates the importance of] champion coherence, external and internal relationship building, and the strategic management of a project's organisation-level visibility' (2015: 1)
Lyon et al	Qualitative study to assess barriers to ERAS implementation, conducted at post-operative stage	Colorectal surgery	Qualitative semi-structured interviews. Grounded theory analysis.	18 interviews with MDT staff	Australia	Conclusion: there are four key areas that present barriers to successful ERAS implementation: (i) patient-related factors, (ii) staff-related factors, (iii) practice-related issues and (iv) resources. For ERAS to be implemented successfully and function efficiently with high levels of compliance, these key areas need to

For peer review only

						be addressed (ideally) before launching an ERAS programme, and then carefully managed throughout
Berthelsen et al	Qualitative study to illuminate orthopaedic nurses' perceptions and experiences of providing individual nursing care for older patients in standardised fast-track programmes	Orthopaedic surgery (hip and knee replacement)	Semi-structured interviews. Manifest and latent content analysis	10 interviews with orthopaedic nurses	Denmark	Conclusion: nurses felt they had to compromise their nursing care and ethics in order to comply with the fast-track programme and implement the standardised care that it recommends

For peer review only - http://bmjopen.bmj.com/site/about/guidelines.xhtml

The sample sizes ranged from 8 to 63. The studies focus on the implementation and delivery of ERAS across a variety of clinical contexts: four on colorectal surgery[19,20,21,22], one on gastrointestinal surgery[23], one on abdominal hysterectomy[24], and two on orthopaedics[25]. Participants included in the studies were a wide range of Multidisciplinary Team and Allied Health Professional staff, and therefore incorporate a diverse range of clinical and professional perspectives. These include registrars, consultants, surgeons, anaesthetists, doctors, nurses and physiotherapists, as well as nursing managers, ERAS coordinators, care coordinators and service improvement coordinators[21]. Participants in one study were recruited specifically because of their role as local ERAS champions[20]. Individual semi-structured interviews were used for data collection in all eight studies. Two studies conducted focus groups as well as interviews[24], and one also collected and analysed memos and reflective journals completed by participants[23]. The different methodologies used in the included studies emphasise the usefulness of this review in drawing together a range of perspectives on staff experiences of implementing ERAS programmes.

The included studies incorporated data gathered at various stages of ERAS implementation: before, during and after. Studies 20, 22 and 26 include information about staff experiences of ERAS pre-implementation and identify their areas of concern about potential barriers (e.g. limited local resources and resistance to change) prior to the introduction of the programmes. These studies, along with study 19, also incorporate data from the peri- and post-implementation stages of ERAS. They show that, despite the presence of such barriers, ERAS programmes were perceived as having brought about changes for the better, even where this process had been challenging. Studies 21 and 23 focus on the post-implementation stage of ERAS, and reflect on the various challenges described by staff, making suggestions for possible improvements. Gotlib Conn et al [20] provide a unique perspective, given that the implementation of ERAS constitutes part of the study, thereby encompassing the experiences of staff champions throughout the entire implementation process. It therefore explores the success and sustainability of ERAS in both the shorter and longer term from the champions' perspective. Despite their different contexts, stages of ERAS implementation and

For peer review only - http://bmjopen.bmj.com/site/about/guidelines.xhtml

surgical populations, the findings from the included studies were largely consistent with one another.

Analysis yielded four themes which are shown in Table 1: communication and collaboration, resistance to change, role and significance of protocol-based care, and knowledge and expectations. The themes identify the key elements of health professionals' experiences of and perspectives on participation in an ERAS pathway. Each theme is described in turn.

Theme 1: Communication and collaboration

Findings from all of the studies emphasised that the successful integration of ERAS practices depends upon effective multidisciplinary team (MDT) communication, and a shared willingness to collaborate. Where this worked well, comprehensive education for staff and patients about ERAS, as well as clear and effective dissemination of knowledge and information were felt to be contributing factors. The high turnover of MDT staff was cited as presenting a challenge to this process, and it was suggested that providing a 'thorough introduction'[24] about ERAS principles to new staff helped to improve matters. Good team work was also seen to be crucial[22], since this helped to foster an environment in which discipline or intervention specific concerns[19], and issues relating to staff and practice[21] could be addressed. Strong team communication was also seen as a means of mitigating staff confusion about ERAS[21]: specific areas identified as requiring improvement were communications between nurses and surgeons[19], dialogue between staff and patients, in which the compressed and information-filled approach of ERAS can prove especially challenging[24]. Having a small clinical community and a close-knit team was recognised as creating a good basis for effective organisational interactions[22].

Staff also drew attention to the challenges of coordinating the various aspects of the ERAS programme, and maintaining a good collaborative approach to this within the MDT[23]: indeed,

For peer review only - http://bmjopen.bmj.com/site/about/guidelines.xhtml

there were concerns that a lack of coordination across different clinical departments served to jeopardise ongoing consistency of practice[22], and it was felt that the provision of feedback and audits to hospital stakeholders[20] was a valuable communicative resource in this respect.

For staff working as champions, building good relationships in and across participating ERAS centres was essential for the successful integration of the programme. They recognised that such relationships served to encourage communication about - and, thereby, establish better shared understandings of current practices on the ground[20], and raise awareness about ERAS guidelines[22] by making sure that everyone is onboard. It was felt that ERAS programmes were most effectively introduced using a bottom up, as opposed to a top down approach[20]. Champions indicated that staff were more likely to engage positively with the integration of ERAS practices where they are able to be involved in co-creating them from the ground up, since this collaborative endeavour helped to foster a collective sense of responsibility[20].

Theme 2: Resistance to change

Data from the studies included in this review highlighted how *resistance to change* amongst staff had presented a major challenge to the implementation of ERAS at both collective and individual levels. It was noted, for instance, that introducing and implementing the programme requires a culture change[19,20] for staff, which they expect to find big and dramatic[23]. Concerns about the unfamiliarity of new working practices can lead to negative attitudes and a reluctance to engage with ERAS guidelines[23], whilst a fundamental dislike of change more widely also provokes disinclination[19,22]. The scope and intensity of the resistance described here is also motivated by staff age and experience[21]. Newer nurses, for instance, found it easier to adjust to the programme and tended to do so more quickly that those who were seen to be stuck in old ways[23].

Appointing a “champion” was recognised as having been extremely helpful in terms of encouraging positive attitudes and effective collaboration when implementing ERAS programmes[19,20,26]. Staff

For peer review only - http://bmjopen.bmj.com/site/about/guidelines.xhtml

taking up this, or a similar, role were appointed from a range of MDT disciplines, and included a ward based designated ERAS nurse[23] and an ERAS coordinator[21]. From the perspective of the champions themselves, meanwhile, resistance was conceptualised less broadly and in more precise terms: attributing this, for instance, to a lack of agreement about specific interventions rather than wider processes[20]. They also felt that even where MDTs were, on the whole, easily accepting of ERAS guidelines, there could still be individual level resistance[20] from some staff.

Theme 3: Role and significance of protocol based care

Staff recognised that working to evidence based guidelines and related protocols can in principle be helpful, because doing so ‘’provides a framework to optimise patient flow by examining what should be done, when, and by whom, thereby reducing delays for patients’’[23: p.30] standardising practices, reducing variations in treatment, and thereby ostensibly improving the quality of patient care. In practice, however, there were mixed feelings amongst MDT staff as to whether or not this was the case in relation to delivering ERAS interventions. Surgeons felt that these were easily implementable as long as they were based on best evidence and incorporated in standardised order sets[19], whilst anaesthesiologists acknowledged that although they were not currently following a standardised protocol, they were open to the idea of implementing standardised guidelines[19]. There was also agreement amongst MDT staff that the implementation of the ERAS programme would provide consistency across working practices[22].

The studies highlighted several challenges of “fittingness” in relation to ERAS programmes, emphasising the relevance of institutional, organisational and patient factors. Champions noted that ERAS pathways are not definitively prescriptive, and that this leads to variability in how they ultimately become integrated into and operationalised within a site’s existing clinical systems[20]. One study found that needing to modify or deviate from ERAS protocols could create confusion for staff[21]. Difficulties in fitting high numbers of patients into the timescales recommended for length

For peer review only - http://bmjopen.bmj.com/site/about/guidelines.xhtml

of hospital stay under ERAS were also cited as a challenge. Nursing staff seemed to experience the greatest impact of these particular challenges on their day to day work, in which they were faced with the reality that some patients do not and cannot comply with ERAS requirements and do not "fit" standard care trajectories, because they are too frail and old, or have very high levels of comorbidity, and are simply too unwell[21,25]. Such issues presented ethical as well as logistical difficulties for nursing staff. Some described feeling highly conflicted about the tensions they experienced in striving to achieve the standardised care targets of ERAS protocols whilst also upholding their ideals of nursing practice[25]. They felt that they were having to make compromises in their work, and experienced this as a struggle. Particular concerns were raised about the detrimental impact that this was having upon nurses' capacity for providing adequately individualised care for patients[25], and the notion of having one protocol for all[23] was felt to be unsatisfactory. Nursing staff felt that the absence of clear guidance about when and how to default or deviate from ERAS protocols led them to be overly cautious in their work, and they indicated that better defined and more precise inclusion criteria about which patients to drive through recovery would be helpful[23].

Theme 4: Knowledge and expectations

Staff recognised that a good knowledge and understanding of ERAS is crucial if it is to be successfully implemented, although the scope of this requirement transcends the procedural details and pragmatic instructions provided by ERAS protocols themselves. Rather, it was important for staff to have a good grasp of its wider aims and objectives[23], and to believe in the value and (potentially) positive impact of the intervention[20,23]. Three of the studies found that, on the whole, staff did feel positive about and favourable towards the implementation of ERAS[19,20,22], and one study showed that although staff were sceptical about it prior to implementation, they felt more positive having seen how well ERAS worked in practice[24]. In all the studies, however, staff acknowledged that considerable challenges still exist and that these will need to be overcome. The nature of such

For peer review only - http://bmjopen.bmj.com/site/about/guidelines.xhtml

concerns varied for staff, depending upon their own MDT specialty, since this had impact upon the way in which they engaged with ERAS practices in their everyday work. Nurses, in particular, described feeling cautious and sceptical about implementing ERAS because of a lack of confidence, indecision, and anxieties about being challenged by other members of the MDT during ward rounds. They were also worried about any potentially adverse consequences for patients of progressing their recovery in accordance with ERAS[23]. Tacit knowledge was also understood to be important for nurses for their role in implementing ERAS: this helped them to take a common sense[23] approach to the process, especially in terms of knowing when it was appropriate to deviate from ERAS guidelines[23,25].

Setting and effectively managing expectations was a key concern for health professionals in helping them to build shared understandings around ERAS, and to understand their own individual tasks and responsibilities. The expectations of both professionals and patients (and negotiations of the two) were relevant here. Staff felt that they themselves benefitted from setting clear patient expectations[19], and were also keenly aware of some of the complex difficulties in collective understandings of what was expected from whom, when, and in which ways across the MDT, where various parties ‘’made an effort to fulfil the other’s expectations in the situation, but from different perspectives and different understandings of the same situation’’[24: p.420].

Pearsall et al.[19] note that staff expectations - of self and others - differ across the MDT and, importantly, explore how these are linked to (sometimes incorrect) assumptions made by some staff about the knowledge and expectations of their colleagues, creating uncertainty and ambivalence around ERAS implementation. For instance, where nurses anticipated that some surgeons might resist ERAS recommendations, surgeons thought that nursing culture and lack of nursing time would present a problem. Anaesthetists, meanwhile, were concerned that patients would not understand ERAS guidelines and procedures, and assumed that it would be very difficult to amend existing and

For peer review only - http://bmjopen.bmj.com/site/about/guidelines.xhtml

well-established nursing culture and surgeon behaviours. The surgeons themselves were unconvinced as to whether changes made in accordance with ERAS would make any difference to patients' experiences of the surgical pathway.

Staff acknowledged that their expectations about ERAS timeframes should be realistic[23], that is, accepting of the reality that some patients would be unable to achieve recovery according to the goals prescribed in the protocol. Whilst some nurses conceptualised such non-achievement as a failure of the [ERAS] programme[23], however, others saw the patients themselves as being responsible for this, on account of them being unprepared for a short hospital stay or early mobilisation, and feeling disproportionately anxious about the process[25]. Staff recognised the extent to which good pre-operative education is helpful for patients, but noted that they nevertheless have to deal with problems arising where patients have unrealistic expectations, forget important information, or simply will not comply with ERAS instructions[21]. It was also felt that some patients might be unable to understand the information and instructions that they received, creating difficulties for MDT staff[22].

For peer review only

DISCUSSION

Our meta-synthesis of qualitative studies produced four themes, which reflect key considerations described by health professionals in relation to their experiences of delivering ERAS pathways. These themes were communication and collaboration, resistance to change, role and significance of protocol-based care and knowledge and expectations. Staff emphasised that there must be effective MDT collaboration and communication, if ERAS practices are to be successfully implemented and integrated. This included providing a thorough education to staff and patients about ERAS, and

For peer review only - http://bmjopen.bmj.com/site/about/guidelines.xhtml

ensuring that information and knowledge about it was clearly and consistently disseminated across the MDT. The coordination of ERAS approaches was acknowledged to be challenging, and the appointment of a designated ERAS champion was experienced as being helpful in this respect.

The value of evidence based guidelines was described as useful means of helping to improve patient care by bringing about a standardisation of practices and a reduction in variations in treatment, but staff were ambivalent about the extent to which ERAS created such consistencies in practice. Concerns were raised about the necessity of modifying or deviating from ERAS guidelines, where these did not “fit” with local site systems or with the care requirements of individual patients. A need for more precise information about how best to do this was identified.

A comprehensive knowledge and understanding of ERAS was cited as being essential to its successful implementation: in terms of both procedural detail and the broader aims and objectives that underpin the intervention itself. Staff were concerned about the impact of ERAS upon their own everyday working practices, and in relation to their own speciality within the MDT. Staff expectations about ERAS varied across MDT disciplines, and the need to set and manage these effectively was prioritised. The importance of establishing ‘realistic’ expectations was emphasised for staff and also the patients for whom they care. This is a key finding that underpins the need for clear guidance to staff who are delivering ERAS.

The implementation and embedding of ERAS was understood to require complex processes of adjustment, acceptance and engagement for staff, constituting a process that evolves gradually over time. Staff attitudes towards ERAS were also subject to temporal change, and tended to become increasingly favourable via reflections upon how well the new and or amended practices were working, and the ways in which they became ‘normalised’.

For peer review only

For peer review only - http://bmjopen.bmj.com/site/about/guidelines.xhtml

Given that ERAS seeks to improve patients' outcomes through consistency in care, findings from our review highlight that, whilst health professionals are confident that ERAS pathways have the potential to achieve this, some key improvements are needed. The findings of this review are new because they highlight key and common themes that appear in all delivery of ERAS in diverse contexts. They also build upon existing knowledge about ERAS by showing that the pathway is implemented disparately across different settings, according to local contexts and circumstances[4], and that the provision of better information and education to staff and patients can achieve better consistency. Our review also indicates that health professionals cite resistance to change amongst staff as a hindrance to the effective implementation of ERAS[6], Our findings demonstrate that effective collaboration and communication amongst staff – and between staff and patients – helps to improve the effectiveness of ERAS[5] and, again, good clear guidance could help with this. The most important finding from the included studies is that appointing a dedicated Enhanced Recovery "champion" is helpful in mitigating many of the barriers to the effective implementation of ERAS [7]. Existing literature finds that champions are central to the successful implementation of complex interventions and practice changes in healthcare settings [27] and that they play a key role in quality improvement when new programmes are introduced [28, 29]. The studies included in our review indicate that the presence of an ERAS champion improves MDT communication and collaboration, assists the provision of consistent information and education to staff and patients, and helps to alleviate resistance to change and lack of confidence amongst staff when they are faced with new working practices brought about by ERAS protocols. Their enthusiastic promotion of new working practices improves staff confidence and skills at a local level, thereby helping to overcome resistance to change [30]. The is the key implication of this review, and an important message for future practice.

We conducted the systematic review in a manner that was designed to capture as many studies as possible by using keywords that were identified and refined from existing literature. To enhance

For peer review only - http://bmjopen.bmj.com/site/about/guidelines.xhtml

rigour in study selection the included studies were all appraised by the two authors. This process acted as a screening process that allowed us to exclude three studies and retain eight as well as appraise whether the included studies sufficiently addressed the ten questions from the CASP qualitative checklist. Assessment using the CASP checklist can be conducted in a variety of ways and our process enabled us to define all studies to be of sufficient quality. To improve reporting quality of this review, we have adhered to the ENTREQ guidance on the reporting of qualitative syntheses[31]. The reflexive approach of the authors in the selection process sought to minimise researcher bias.

One of the strengths of this review is that it includes a range of different studies, and therefore incorporates a variety of populations and geographical contexts. Further strengths are the diversity of methodological approaches used in the studies, and the different clinical contexts and local environments of the included studies. This provides a richness of perspectives. This paper therefore makes a valuable contribution to the field of literature. A limitation of this review is the small number of included studies, however, we included studies in six countries across four surgical specialities and as such our work highlights key issues that are transferable between contexts. Tere are no ethnographic studies included in our review, and we suggest that future research could build upon existing knowledge of and understanding about staff perspectives of ERAS by taking an ethnographic approach. The value of using qualitative ethnographic study in healthcare settings is well documented [32,33,34]. The findings from this review indicate that an ethnographic approach would enable a more nuanced understanding of the ways in which care pathways are organised, explained, understood, performed and delivered across different hospital contexts and settings, and to contrast and compare elements of care and practice. We also note that ERAS pathways are now being implemented in elective orthopaedic surgery, and suggest that this is a valuable area for future study.

For peer review only - http://bmjopen.bmj.com/site/about/guidelines.xhtml

CONCLUSION

We reviewed and synthesised qualitative studies that explore health professionals' experiences of and perspectives on the enhanced recovery after surgery (ERAS) pathway. This is the first systematic review to draw together findings from qualitative studies with health professionals, and to inform implementation of ERAS we would argue that their experiences and views are crucial. Findings from our review indicate that, whilst staff generally feel positive about the implementation of ERAS, they acknowledge that the process is complex and challenging. Many of the challenges identified, such as resistance to change and lack of confidence can however be mitigated by ensuring that MDTs understand ERAS principles and guidelines, and that they communicate well with one another and with patients. Other challenges, such as a lack of local resources and high rates of comorbidity amongst patients are perhaps more challenging to address. We suggest that the provision of comprehensive, coherent and locally relevant information to health professionals would help to improve the implementation and delivery of ERAS pathways. Identifying and recruiting an ERAS champion is also recommended as means of improving the effectiveness of the pathway.

A: CONTRIBUTORSHIP STATEMENT

The authors of this article are Dr Rachel Cohen (RC) and Professor Rachael Gooberman-Hill (RGH). Both authors made substantial contributions to the conception and the design of the systematic review. Literature searches were conducted by RC, and RGH carried out the CASP screening. Both RC and RGH contributed to the extraction, analysis and interpretation of data from the papers included in the review. RC and RGH worked on drafts of the review, made revisions and agreed on a final version for publication. Both RC and RGH agree to be accountable for all aspects of the work in ensuring that questions related to the accuracy or integrity of any part of the work are appropriately investigated and resolved.

For peer review only - http://bmjopen.bmj.com/site/about/guidelines.xhtml

For peer review only

B: COMPETING INTERESTS

The author and co-author have no competing interests to declare

C: FUNDING

This review forms part of the dissemination strategy for the Atlas (Ethnographic study of care pathways for hip and knee replacement) project, which is funded by the National Institute for Health Research Health Services and Delivery Research Programme (project number 14/46/02).

For peer review only

D: DATA SHARING STATEMENT

Data for the study may be made available from University of Bristol's research data repository under a controlled access arrangement. Requests for access will be referred to the University's data access committee before data can be shared under a data sharing agreement. As such, anonymous data from the study may be seen and used by other researchers, for ethically approved research projects, on the understanding that confidentiality will be maintained. Release of the data will be at the discretion of the data access committee (data custodian).

E: ACKNOWLEDGEMENT

Support for the study was received from the Oxford NIHR Biomedical Research Centre, Nuffield Orthopaedic Centre, University of Oxford. The views and opinions expressed therein are those of the authors and do not necessarily reflect those of the HS&DR Programme, NIHR, NHS or the Department of Health. We thank the wider team for the study, including Professor Andrew Judge as Principal Investigator. We also thank Christine Hobson for her work in support of our research.

For peer review only - http://bmjopen.bmj.com/site/about/guidelines.xhtml

For peer review only

REFERENCES

1. Kehlet H. Fast-track colorectal surgery. *The Lancet* 2008;371:791-3.

2. Department of Health. Enhanced Recovery Partnership Programme Project Report. March 2011.Available from: https://www.gov.uk/government/publications/enhanced-recovery-partnership-programme (accessed 1 Feb 2017).

3. Campbell NC, Murray E, Darbyshire J, *et al*. Designing and evaluating complex interventions to improve health care. *BMJ* 2007;334:455-9.

4. Craig P, Dieppe P, Macintyre S, *et al.* Developing and Evaluating Complex Interventions: The New Medical Research Council Guidance. *BMJ* 2008;337:979-83.

For peer review only - http://bmjopen.bmj.com/site/about/guidelines.xhtml

5. Waring J, Marshall F, Bishop S, *et al*. An ethnographic study of knowledge sharing across the boundaries between care processes, services and organisations: the contributions to 'safe' hospital discharge. *Health Serv Deliv Res* 2014:2;1-160.

6. Moore G, Audrey S, Barker M, *et al*. Process evaluation in complex public health intervention studies: the need for guidance. *J Epidemiol Community Health* 2014;68:101-2.

7. Paton F, Chambers D, Wilson P, *et al.* Effectiveness and implementation of enhanced recovery after surgery programmes: a rapid evidence synthesis. *BMJ Open* 2014;4:e005015. doi:10.1136/bmjopen-2014-005015.

8. Bernard H, Foss M. The impact of the enhanced recovery after surgery (ERAS) programme on community nursing. *Br J Community Nurs* 2014;19:184-8.

9. Blazeby JM, Soulsby M, Winstone K, *et al*. A qualitative evaluation of patients' experiences of an enhanced recovery programme for colorectal cancer. *Colorectal Dis* 2010;12:e236-42.

10. Hunt GR, Hall GM, Murthy BV, *et al*. Early Discharge Following hip Arthroplasty: Patients' acceptance masks doubts and concerns. *Health Expect* 2009;12:130-7.

11. Husted H, Holm G. Fast track in total hip and knee arthroplasty - experiences from Hvidovre University Hospital, Denmark. *Injury* 2006;37(Suppl 5):S31-S35.

12. Norlyk A. The experience of fast track postoperative rehabilitation regimen: the patients' perspective. *Klinisk Sygepleje* 2008;22:53-63.

For peer review only - http://bmjopen.bmj.com/site/about/guidelines.xhtml

For peer review only

13. Norlyk A, Harder I. After colonic surgery: The lived experience of participating in a fast-track programme. *Int J Qual Stud on Health Well-being* 2009;4:170-180.

14. Sibbern T, Bull Sellevold V, Steindal SA, *et al*. Patients' Experiences of Enhanced Recovery after Surgery: a systematic review of qualitative studies. *J Clin Nurs* 2017;26:1172-188.

15. Critical Skills Appraisal Programme. *CASP Qualitative Checklist*. Available from: http://www.casp-uk.net/checklists (accessed 30 May 2018).

16. Polit DF, Beck CT. Nursing Research: Generating and Assessing Evidence for Nursing Practice. Philadelphia: Wolters Kluwer/Lippincott Williams and Wilkins; 2008.

17. Graneheim UH, Lundman B. Qualitative content analysis in nursing research: concepts, procedures and measures to achieve trustworthiness. *Nurse Educ Today* 2004;24:105–12.

18. Uhrenfeldt L, Aagaard H, Hall EO, *et al.* A qualitative meta-synthesis of patients' experiences of intra- and inter-hospital transitions. *J Adv Nurs* 2013;69:1678–90.

19. Pearsall EA, Meghiji A, Pitzul KB, *et al*. A Qualitative Study to Understand the Barriers and Enablers in Implementing an Enhanced Recovery after Surgery Program. *Ann Surg* 2015;261:92-6.

20. Gotlib Conn L, McKenzie M, Pearsall EA, *et al*. Successful implementation of an enhanced recovery after surgery programme for elective colorectal surgery: a process evaluation of champions' experiences. *Implement Sci* 2015;10:99.

For peer review only - http://bmjopen.bmj.com/site/about/guidelines.xhtml

21. Lyon A. Solomon MJ, Harrison JD. A Qualitative Study Assessing the Barriers to Implementation of Enhanced Recovery after Surgery. *World J Surg* 2014;38:1374-80.

22. Alawadi ZM, Leal I, Phatak UR, *et al*. Facilitators and barriers of implementing enhanced recovery in colorectal surgery at a safety net hospital: A provider and patient perspective. *Surgery* 2016;159:700-12.

23. Jeff A, Taylor C. Ward nurses' experiences of enhanced recovery after surgery: a grounded theory approach. *Gastrointestinal Nursing* 2014;12:23-31.

24. Wagner L, Carlslund AM, Moller C, *et al.* Patient and staff (doctors and nurses) experiences of abdominal hysterectomy in accelerated recovery programme: A qualitative study. *Dan Med Bull* 2004;51:418-21.

25. Berthelsen CB, Frederiksen K. Orchestrating care through the fast track perspective: A qualitative content analysis of the provision of individualised nursing care in orthopaedic fast-track programmes. *Int J Orthop Trauma Nurs* 2017;24:40-9.

26. Sjetne IS, Krogstad U, Odegard, S, *et al*. Improving quality by introducing enhanced recovery after surgery in a gynaecological department: consequences for ward nursing practice. Qual Saf Health Care; 2009:18;236-40.

27. Soo SD, Berta W, Baker GR. The role of champions in the implementation of patient safety practice change. *Healthcare Quarterly*; 2009: 12; 123-8

For peer review only

For peer review only - http://bmjopen.bmj.com/site/about/guidelines.xhtml

28. Raval MV, Bentrem DJ, Eskandari MK, *et al*. The role of surgical champions in the American College of Surgeons National Surgical Quality Improvement Program – a national survey. *J. Surg Res;* 2011; 16: 15-25

29. Sartelli M, Kluger Y, Ansaloni L, *et al.* Knowledge awareness and attitude towards infection prevention and management among surgeons: identifying the surgeon champion. *World J Emerg Surg;* 13: 37

30. Shaw EK, Howard J, West DR, *et al.* The role of the champion in primary care change efforts*. J Am Board Fam Med*; 2012: 25; 676-685

31. Tong A, Flemming K, McInnes *et al*. Enhancing transparency in reporting the synthesis of qualitative research: ENTREQ. *BMC Med Res Methodol* 2012;12:181.

32. Mays, N, Pope C. Observational methods in healthcare settings. *BMJ* 1995; 311

33. Bloor M. On the analysis of observational data: a discussion of the worth and uses of inductive techniques and respondent validation. *Sociology* 1978; 12: 545-52

34. Savage J. Ethnography and healthcare*. Br Med J* 2000; 321:1400-2

For peer review only - http://bmjopen.bmj.com/site/about/guidelines.xhtml

PRISMA 2009 Flow Diagram
Staff experiences of Enhanced Recovery after Surgery – Systematic Review of Qualitative Studies

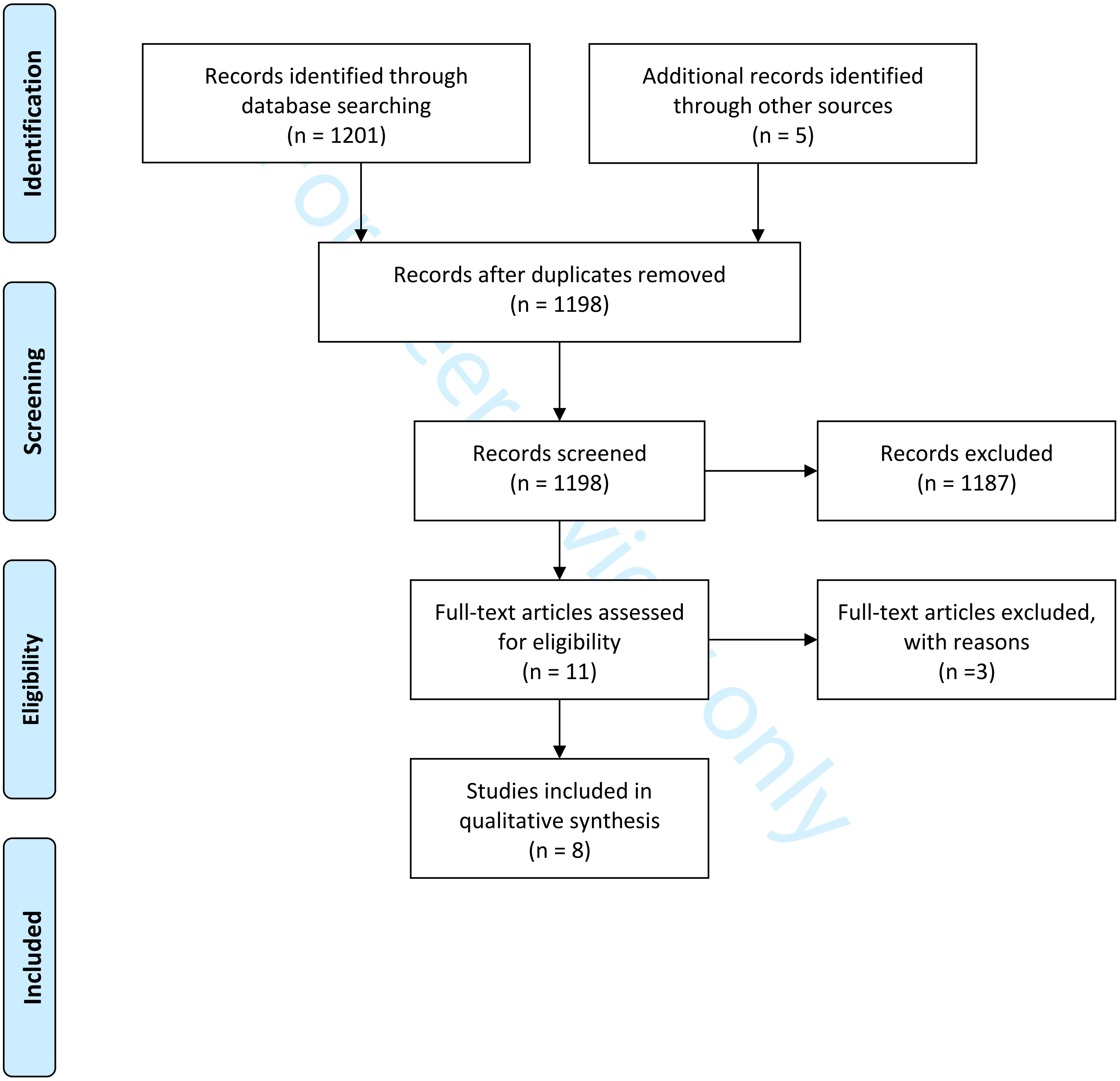

From: Moher D, Liberati A, Tetzlaff J, Altman DG, The PRISMA Group (2009). *P*referred *R*eporting *I*tems for *S*ystematic Reviews and *M*eta-*A*nalyses: The PRISMA Statement. PLoS Med 6(7): e1000097. doi:10.1371/journal.pmed1000097

For more information, visit www.prisma-statement.org.

For peer review only - http://bmjopen.bmj.com/site/about/guidelines.xhtml

PRISMA 2009 Checklist

Section/topic	#	Checklist item	Reported on page #
**TITLE**			
Title	1	Identify the report as a systematic review, meta-analysis, or both.	1
**ABSTRACT**			
Structured summary	2	Provide a structured summary including, as applicable: background; objectives; data sources; study eligibility criteria, participants, and interventions; study appraisal and synthesis methods; results; limitations; conclusions and implications of key findings; systematic review registration number.	2
**INTRODUCTION**			
Rationale	3	Describe the rationale for the review in the context of what is already known.	4
Objectives	4	Provide an explicit statement of questions being addressed with reference to participants, interventions, comparisons, outcomes, and study design (PICOS).	5
**METHODS**			
Protocol and registration	5	Indicate if a review protocol exists, if and where it can be accessed (e.g., Web address), and, if available, provide registration information including registration number.	5
Eligibility criteria	6	Specify study characteristics (e.g., PICOS, length of follow-up) and report characteristics (e.g., years considered, language, publication status) used as criteria for eligibility, giving rationale.	6
Information sources	7	Describe all information sources (e.g., databases with dates of coverage, contact with study authors to identify additional studies) in the search and date last searched.	5,6
Search	8	Present full electronic search strategy for at least one database, including any limits used, such that it could be repeated.	5,6
Study selection	9	State the process for selecting studies (i.e., screening, eligibility, included in systematic review, and, if applicable, included in the meta-analysis).	5,6,7
Data collection process	10	Describe method of data extraction from reports (e.g., piloted forms, independently, in duplicate) and any processes for obtaining and confirming data from investigators.	6-9
Data items	11	List and define all variables for which data were sought (e.g., PICOS, funding sources) and any assumptions and simplifications made.	6-9
Risk of bias in individual studies	12	Describe methods used for assessing risk of bias of individual studies (including specification of whether this was done at the study or outcome level), and how this information is to be used in any data synthesis.	6-9
Summary measures	13	State the principal summary measures (e.g., risk ratio, difference in means).	N/A
Synthesis of results	14	Describe the methods of handling data and combining results of studies, if done, including measures of consistency (e.g., I^2) for each meta-analysis.	6-9

For peer review only - http://bmjopen.bmj.com/site/about/guidelines.xhtml

PRISMA 2009 Checklist

Section/topic	#	Checklist item	Reported on page #
Risk of bias across studies	15	Specify any assessment of risk of bias that may affect the cumulative evidence (e.g., publication bias, selective reporting within studies).	N/A
Additional analyses	16	Describe methods of additional analyses (e.g., sensitivity or subgroup analyses, meta-regression), if done, indicating which were pre-specified.	N/A
**RESULTS**			
Study selection	17	Give numbers of studies screened, assessed for eligibility, and included in the review, with reasons for exclusions at each stage, ideally with a flow diagram.	6,7
Study characteristics	18	For each study, present characteristics for which data were extracted (e.g., study size, PICOS, follow-up period) and provide the citations.	10-12
Risk of bias within studies	19	Present data on risk of bias of each study and, if available, any outcome level assessment (see item 12).	N/A
Results of individual studies	20	For all outcomes considered (benefits or harms), present, for each study: (a) simple summary data for each intervention group (b) effect estimates and confidence intervals, ideally with a forest plot.	N/A
Synthesis of results	21	Present results of each meta-analysis done, including confidence intervals and measures of consistency.	N/A
Risk of bias across studies	22	Present results of any assessment of risk of bias across studies (see Item 15).	N/A
Additional analysis	23	Give results of additional analyses, if done (e.g., sensitivity or subgroup analyses, meta-regression [see Item 16]).	N/A
**DISCUSSION**			
Summary of evidence	24	Summarize the main findings including the strength of evidence for each main outcome; consider their relevance to key groups (e.g., healthcare providers, users, and policy makers).	19-21
Limitations	25	Discuss limitations at study and outcome level (e.g., risk of bias), and at review-level (e.g., incomplete retrieval of identified research, reporting bias).	21,22
Conclusions	26	Provide a general interpretation of the results in the context of other evidence, and implications for future research.	21,22
**FUNDING**			
Funding	27	Describe sources of funding for the systematic review and other support (e.g., supply of data); role of funders for the systematic review.	23

From: Moher D, Liberati A, Tetzlaff J, Altman DG, The PRISMA Group (2009). Preferred Reporting Items for Systematic Reviews and Meta-Analyses: The PRISMA Statement. PLoS Med 6(7): e1000097. doi:10.1371/journal.pmed1000097

For more information, visit: **www.prisma-statement.org**.

For peer review only - http://bmjopen.bmj.com/site/about/guidelines.xhtml
